# A new mathematical model of phyllotaxis to solve the genuine puzzle spiromonostichy

**DOI:** 10.1007/s10265-023-01503-2

**Published:** 2023-10-13

**Authors:** Takaaki Yonekura, Munetaka Sugiyama

**Affiliations:** Department of Biological Sciences, Graduate School of Science, The University of Tokyo, Hongo 7-3-1, Bunkyo-ku, Tokyo, 113-0033 Japan

**Keywords:** *Costus*, Mathematical modeling, Pattern formation, Phyllotaxis, Spiromonostichy

## Abstract

Arrangement of plant leaves around the stem, termed phyllotaxis, exhibits beautiful and mysterious regularities and has been one of the most attractive subjects of biological pattern formation. After the long history of studies on phyllotaxis, it is now widely accepted that the inhibitory effect of existing leaf primordia on new primordium formation determines phyllotactic patterning. However, costoid phyllotaxis unique to Costaceae of Zingiberales, displaying spiromonostichy characterized by a steep spiral with a small divergence angle, seems to disagree with the inhibitory effect-based mechanism and has remained as a “genuine puzzle”. We developed a new mathematical model, hypothesizing that each leaf primordium exerts not only the inhibitory effect but also some inductive effect. Computer simulations with the new model successfully generated a spiromonostichous pattern when these two effects met a certain relationship. The obtained spiromonostichy matched the real costoid phyllotaxis observed with *Costus megalobractea*, particularly for the decrease of the divergence angle associated with the enlargement of the shoot apical meristem. The new model was also shown to be able to produce a one-sided distichous pattern that is seen in phyllotaxis of a few plants of Zingiberales and has never been addressed in the previous model studies. These results implicated inductive as well as inhibitory mechanisms in phyllotactic patterning, at least in Zingiberales.

**Supplementary Information:**

The online version contains supplementary material available at 10.1007/s10265-023-01503-2.

## Introduction

Phyllotaxis is one of the most conspicuous patterns seen in plant architecture because of its geometric beauty. There are various types of phyllotaxis but the variety is rather limited. Most plant species show one of the four common types of phyllotaxis: distichous, Fibonacci spiral, decussate, and tricussate (Yonekura and Sugiyama 2021), where Fibonacci spiral represents spiral phyllotaxis with the divergence angle close to the golden angle, 137.5°, and with a mathematical relation to the Fibonacci sequence (Jean 1994). The limited variation suggests some constraints coming from the regulatory mechanism behind phyllotactic pattern formation. In this respect, Hofmeister described an empirical law of leaf primordium positioning (Hofmeister 1868) and is now known as Hofmeister’s axiom (Jean 1994). It states that, on the periphery of the shoot apical meristem (SAM), “each leaf arises in the largest gap between existing leaves or primordia and as far away as possible from them” (Jean 1994).

Following Hofmeister’s axiom, various models assuming some kind of repulsive interaction between leaf primordia were proposed to explain phyllotactic pattern formation (Adler 1974; Douady and Couder 1992, 1996a, b; Levitov 1991; Mitchison 1977; Roberts 1977; Snow and Snow 1962). Among these models, the second model of Douady and Couder (Douady and Couder 1996b), referred to as DC2 hereafter, was most extensively analyzed and has served as a framework for later theoretical studies of phyllotactic pattern formation. DC2 postulates an inhibitory field constantly emanated from the center of each leaf primordium. Primordium initiation occurs whenever and wherever the inhibitory field strength falls below a given threshold on the SAM periphery. Computer simulations with DC2 successfully produced all major types of phyllotaxis.

In the 2000s, physiological and molecular biological studies indicated the critical importance of auxin and its polar transport driven by PIN1 in the initiation of shoot lateral organs (Benková et al. 2003; Reinhardt et al. 2000, 2003). Further analysis suggested that a positive feedback loop between auxin distribution and PIN1 localization works for the spontaneous generation of auxin convergence in the epidermal layer of the shoot apex and these findings were integrated into mathematical models (Jönsson et al. 2006; Smith et al. 2006). In these models, the auxin convergence determines the site of primordium initiation, and auxin depletion into the convergence from its surroundings by the positive feedback dynamics generates the inhibitory field. The parameters of the auxin-based models were mapped to the parameters of DC2, which showed that DC2 can be treated as an abstract model of phyllotactic pattern formation regulated by auxin (Mirabet et al. 2012).

Although DC2 and auxin-based models provided a basis for understanding the formation of major phyllotactic patterns, they did not address several minor types of phyllotaxis such as orixate phyllotaxis. Additionally, these models did not fully account for the overwhelming dominance of Fibonacci spiral in spiral phyllotactic patterns. In an attempt to address these problems, we constructed an expanded version of DC2, which we call EDC2, by introducing a primordial age-dependent increase of the inhibitory field emission (Yonekura et al. 2019). As a result of computer simulation analysis, EDC2 was demonstrated to reproduce broader types of phyllotaxis including orixate phyllotaxis and fit better to the dominance of Fibonacci spiral than the previous models (Yonekura et al. 2019).

**Fig. 1:**
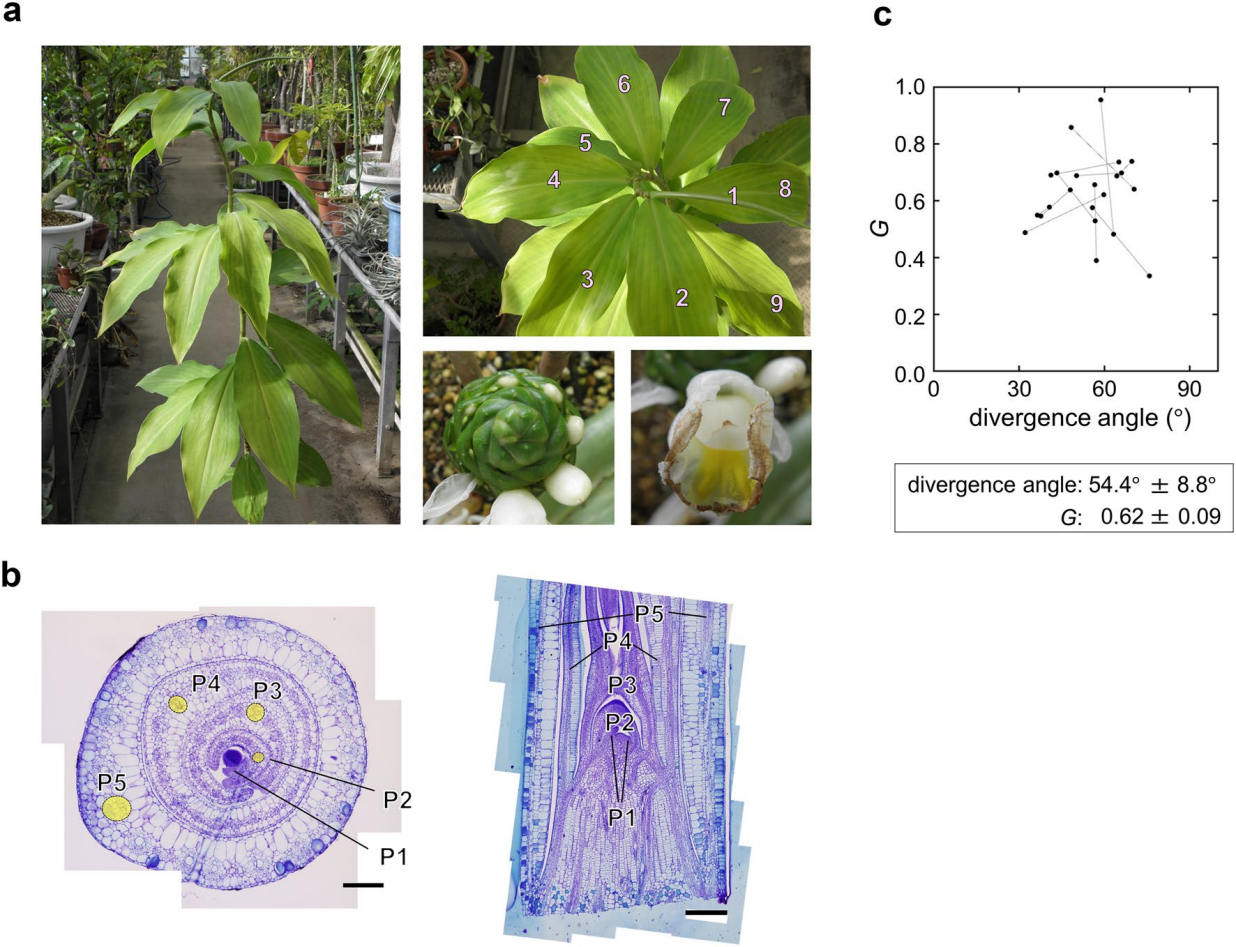
Morphological characteristics of phyllotaxis of *C. megalobractea*. **a**, General views of the shoot, inflorescence, and flower of *C. megalobractea*. **b**, Transverse and longitudinal sections of the vegetative shoot apex of *C. megalobractea*. The youngest visible leaf (primordium) is symbolized by ${P}_{1}$, the next youngest ${P}_{2}$, and so forth. For ${P}_{2}$ to ${P}_{5}$, the midveins are marked with yellow color. Bars: 200 μm. **c**, Divergence angles and $G$ values of ${P}_{1}-{P}_{2}$ and ${P}_{2}-{P}_{3}$ measured using the transverse sections. Points linked by a line represent data from the same sample

Now almost all phyllotactic patterns that occur in nature can be produced by mathematical models. However, there still remain a few exceptions. Of these, the most striking one is costoid phyllotaxis uniquely found in Costaceae, Zingiberales (Fig. 1a). Costoid phyllotaxis is characterized by spiromonostichy, that is a steep spiral with a small divergence angle (Snow 1952) in which all leaves are lined up in a single oblique row, i.e., monostichy. The spiromonostichous appearance of costoid phyllotaxis clearly distinguishes it from common spiral phyllotaxis. It is also notable that the divergence angle of costoid phyllotaxis is continuously variable during development and between species and is not converged to specific values linked to Fibonacci or related sequences (Kirchoff and Rutishauser 1990). The small divergence angle of costoid phyllotaxis apparently results from a peculiar spatial relationship between leaf primordia such that a new leaf primordium is positioned near its immediately preceding primordium. Because this feature violates Hofmeister’s axiom, costoid phyllotaxis or spiromonostichy has been called a “famous and fascinating puzzle” (Kirchoff and Rutishauser 1990) and a “genuine puzzle” (Jean 1994).

For trying to explain the unusual leaf positioning of costoid phyllotaxis, several ideas have been suggested, but they are just intuitive ideas without having been tested either theoretically or experimentally for their validities, and some of them even obviously contradict morphological observations of costoid phyllotaxis (Kirchoff and Rutishauser 1990). For example, Kirchoff and Rutishauser (1990) mentioned foliar helices postulated by Plantefol (1948) for helical lines winding the stem along which hypothetical generating centers at the shoot apex form leaf primordia periodically, and pointed out the possibility that costoid phyllotaxis might be explained by the induction of a new leaf primordium by the youngest existing primordium along the same foliar helix. However, the foliar helix-based concept of Plantefol was criticized as lacking a developmental perspective and also as being problematic in the initial determination of foliar helices (Cutter 1965; Snow 1948; Wardlaw 1956) and therefore has not been generally accepted. A model for systematically relating a wide variety of phyllotactic patterns including the costoid pattern was proposed by Jean (1988), but this is an interpretative model and does not give any insights into the mechanism of phyllotactic patterning. Thus, so far, there have been no mechanistic models addressing the generation of the costoid pattern.

In the present study, we analyzed costoid phyllotaxis of *Costus megalobractea* K.Schum. morphologically, examined the ability of the previous models to produce costoid phyllotaxis, and then constructed a new mathematical model by introducing a hypothetical inductive field that surrounds the existing leaf primordia and acts positively for new primordium initiation. The results obtained indicate that a balance between expansions of the induction and inhibition ranges is critical for the generation of costoid phyllotaxis. Computer simulation with our new model under appropriate parameter settings produced realistic costoid phyllotaxis. Based on these findings, we discuss the plausibility of the new model as a common basis of phyllotactic patterning.

## Materials, methods, and model descriptions

### Plant materials and growth condition

Shoot apices of *C. megalobractea* that had been collected from adult vegetative plants growing in the greenhouse of Koishikawa Botanical Gardens, Graduate School of Science, The University of Tokyo were subjected to morphological analysis of phyllotaxis. For measurement of early changes in the divergence angle and the SAM size, seedlings of *C. megalobractea* cultured at 25 °C under continuous light from white fluorescent lamps were used instead of adult plants.

### Microscopic observation of sections

The shoot apices of *C. megalobractea* were fixed with FAA (5% v/v formalin, 5% v/v acetic acid, 50% v/v ethanol), dehydrated in an ethanol series (50%, 60%, 70%, 80%, 90%, 95%, 99.5%), and finally infiltrated in 100% ethanol. Then they were embedded in Technovit® 7100, cut into 5-µm-thick sections with a rotary microtome, and stained with 0.5% w/v toluidine blue/0.1% w/v sodium carbonate. Images of the sections were assembled using the MosaicJ plugin (https://imagej.net/MosaicJ) of ImageJ 1.49v (https://imagej.nih.gov/). For each leaf (primordium), the gravity center of the midvein if the midvein was obvious, or the gravity center of the whole leaf (primordium) section if otherwise was determined and used as its position for morphometric analysis.

### Microscopic observation of SAM size

The shoot apices of *C. megalobractea* were fixed with a 3:1 (v/v) mixture of ethanol and acetic acid, hydrated in an ethanol series (75%, 50%, 25%, 0%), washed twice with phosphate-buffered saline (0.02% w/v KH_2_PO_4_, 0.02% w/v KCl, 0.29% w/v Na_2_HPO_4_·12H_2_O, 0.8% w/v NaCl), and finally infiltrated in ClearSee (Kurihara et al. 2015). The cleared shoot apices were observed using differential interference contrast microscopy to measure the SAM width, which was defined as the distance between the two ends of the dome of SAM.

### Mathematical modeling in EDC2

The essential points of EDC2 are as follows (Yonekura et al. 2019).

1. The shoot apex is considered as a cone with an apical angle of $\psi$.
2. Each leaf primordium $L$ emits an inhibitory field around it.
3. The inhibitory field emission increases as a function of the primordial age $t$.
4. The inhibitory field strength decreases as a function of the distance $d$.
5. Formation of new primordia is restricted to the SAM periphery represented by the circle $M$ with a distance of ${R}_{0}$ from the conical vertex.
6. When the inhibitory field strength falls below a given threshold ${E}_{th}$ somewhere on $M$, a new primordium is formed at that point at that moment.
7. Primordia move away from the center of the shoot apex with a radial velocity of $V\left(r\right)$ that is proportional to the radial distance $r$ because of the exponential growth of the shoot apex.

Positions on the conical surface are expressed in spherical coordinates $\left(r, \frac{\psi }{2}, \theta \right)$.

Because of assumption 7, the distance from the center of the shoot apex to the $m$th primordium on the conical surface (${r}_{m}$) is expressed with the time after its emergence ${T}_{m}$ and the initial radial velocity ${V}_{0}$ as:

1 $${r}_{m}={R}_{0}{e}^{\frac{{V}_{0}}{{R}_{0}}{T}_{m}}$$

By using ${t}_{m}\equiv {T}_{m}{V}_{0}/{R}_{0}$, a standardized age of the $m$th primordium defined as the product of ${T}_{m}$ and the relative rate of apical growth ${V}_{0}/{R}_{0}$, ${r}_{m}$ is more simply expressed as:

${t}_{m}-{t}_{m+1}$ gives a standardized plastochron and it can be calculated from Eq. 2 as:

2 $${r}_{m}={R}_{0}{e}^{{t}_{m}}$$

3 $${t}_{m}-{t}_{m+1}=\text{ln}\left(\frac{{r}_{m}}{{R}_{0}}\right)-\text{ln}\left(\frac{{r}_{m+1}}{{R}_{0}}\right)=\text{ln}\left(\frac{{r}_{m}}{{r}_{m+1}}\right)={G}_{m}$$

${r}_{m}/{r}_{m+1}$ is a plastochron ratio, an important quantitative index of phyllotaxis (Richards 1951). Thus, the standardized plastochron is equal to the natural log of the plastochron ratio $G$ (Douady and Couder 1996a) and represented by ${G}_{m}$ for the pair of the $m$th and $m+1$th primordia.

The inhibitory field strength $I\left(\theta \right)$ at the position $\left({R}_{0}, \frac{\psi }{2}, \theta \right)$ on $M$ is calculated by summing the inhibitory effects from all preceding primordia ${L}_{1}$ to ${L}_{n-1}$ as follows:

where ${d}_{m}\left(\theta \right)$ is the distance from the $m$th primordium to the position$\left({R}_{0}, \frac{\psi }{2}, \theta \right)$ and ${d}_{0}$ is the maximum distance within which an existing primordium excludes a new primordium. Two functions $E$ and $F$ are defined as:

4 $$I\left( \theta \right) \equiv \sum\limits_{{m = 1}}^{{n - 1}} {E\left( {\frac{{d_{m} \left( \theta \right)}}{{d_{0} }}} \right)F\left( {t_{m} } \right)}$$

5 $$E\left(x\right)\equiv {E}_{th}\frac{{-1+\left(\text{tanh}\alpha x\right)}^{-1}}{{-1+\left(\text{tanh}\alpha \right)}^{-1}}$$

6 $$F\left(t\right)\equiv \frac{1}{1+{e}^{-A\left(t-B\right)}}$$

where $\alpha$, $A$, and $B$ are constants. If $I\left(\theta \right)<{E}_{th}$, a new primordium is placed at the position $\left({R}_{0}, \frac{\psi }{2}, \theta \right)$. Throughout this study, ${E}_{th}=1$.

EDC2 originally has six parameters: $N\equiv \text{sin}\frac{\psi }{2}$, ${R}_{0}$, ${d}_{0}$, $\alpha$, $A$, and $B$. These parameters represent the flatness of the shoot apex, the SAM size, the maximum range of inhibition from one primordium, the steepness of the decline of the inhibitory effect around the threshold, the steepness of the age-dependent increase of the inhibitory field emission, and the timing of age-dependent increase of the inhibitory field emission, respectively. The effects of the parameters ${R}_{0}$ and ${d}_{0}$ on phyllotactic patterning can be summarized into the effect of a single adimensional parameter $\varGamma \equiv \frac{{d}_{0}}{{R}_{0}\sqrt{N}}$, which indicates the ratio of the maximum range of inhibition to the SAM size. Thus, EDC2 is determined by five independent parameters: $N$, $\varGamma$, $\alpha$, $A$, and $B$.

#### New model construction

EDC2 was modified to build a new model, of which the essential points are as follows.

1. The shoot apex is considered as a cone with an apical angle of $\psi$.
2. Each leaf primordium $L$ emits both an inductive field and an inhibitory field around it.
3. Both the inductive and inhibitory field strengths decline dependently on the distance $d$.
4. Both the inductive and inhibitory field emissions increase dependently on the primordial age $t$.
5. The formation of new primordia is restricted to the SAM periphery represented by the circle $M$ with a distance of ${R}_{0}$ from the conical vertex.
6. When the inductive field strength $Y$ rises above a given threshold ${Y}_{th}$ and the inhibitory field strength $S$ falls below a given threshold ${S}_{th}$ somewhere on $M$, a new primordium is formed at that point at that moment.
7. Primordia move away from the center of the shoot apex with a radial velocity that is proportional to the radial distance $r$ due to the exponential growth of the shoot apex.

Assumptions, 5, and 7 are identical between the new model and EDC2. As is the case for EDC2, positions on the conical surface are expressed in spherical coordinates. Definitions of $r_{m}$, $T_{m}$, $V_{0}$, $t_{m}$, and $G_{m}$ are the same as described for EDC2. Equations 1, 2, and 3 can be applied for the new model as well.

The inductive field strength $Y\left(\theta \right)$ and inhibitory field strength $S\left(\theta \right)$ at the position $\left({R}_{0}, \frac{\psi }{2}, \theta \right)$ on $M$ are calculated by summating inductive and inhibitory effects from all preceding primordia, respectively, as follows:

7 $$Y\left(\theta \right)\equiv {\sum }_{m=1}^{n-1}{Y}_{th}{E}_{Y}\left(\frac{{d}_{m}\left(\theta \right)}{{d}_{Y}};{\alpha }_{Y}\right){F}_{Y}\left({t}_{m};{A}_{Y}, {B}_{Y}\right),$$

8 $$S\left(\theta \right)\equiv {\sum }_{m=1}^{n-1}{S}_{th}{E}_{S}\left(\frac{{d}_{m}\left(\theta \right)}{{d}_{S}};{\alpha }_{S}\right){F}_{S}\left({t}_{m};{A}_{S}, {B}_{S}\right).$$

In these equations, ${d}_{m}\left(\theta \right)$ is a distance between the $m$-th primordium and the position $\left({R}_{0}, \frac{\psi }{2}, \theta \right)$, ${d}_{Y}$ and ${d}_{S}$ are the maximum distance within which a single existing primordium induces a new primordium and the maximum distance within which a single existing primordium excludes a new primordium, respectively. $E$ is a distance-dependent factor, which is defined as:

9 $${E}_{Y}\left(x;{\alpha }_{Y}\right)\equiv {x}^{-{\alpha }_{Y}},$$

10 $${E}_{S}\left(x;{\alpha }_{S}\right)\equiv {x}^{-{\alpha }_{S}}.$$

$F$ is a primordial age-dependent factor, which is defined as:

11 $${F}_{Y}\left(t;{A}_{Y}, {B}_{Y}\right)\equiv \frac{1}{1+{e}^{-{A}_{Y}\left(t-{B}_{Y}\right)}},$$

12 $${F}_{S}\left(t;{A}_{S}, {B}_{S}\right)\equiv \frac{1}{1+{e}^{-{A}_{S}\left(t-{B}_{S}\right)}}.$$

If $Y\left(\theta \right)>{Y}_{th}$ and $S\left(\theta \right)<{S}_{th}$, a new primordium is placed at the position $\left({R}_{0}, \frac{\psi }{2}, \theta \right)$. In this study, ${Y}_{th}$ and ${S}_{th}$ are fixed to $1$.

The new model has ten parameters: $N\equiv \text{sin}\frac{\psi }{2}$, ${R}_{0}$, ${d}_{S}$, ${\alpha }_{S}$, ${A}_{S}$, ${B}_{S}$, ${d}_{Y}$, ${\alpha }_{Y}$, ${A}_{Y}$, and ${B}_{Y}$. The first two parameters $N$ and ${R}_{0}$ are the same as in EDC2. The next four parameters characterize the inhibitory effect and are correspondent to ${d}_{0}$, $\alpha$, $A$, and $B$ in EDC2. The last four parameters characterize the inductive effect and represent the maximum range of induction from one primordium, the steepness of the decline of the inductive effect around the threshold, the steepness of the age-dependent increase of the inductive field emission, and the timing of age-dependent increase of the inductive field emission, respectively. The effects of the parameters ${R}_{0}$, ${d}_{S}$, and ${d}_{Y}$ can be summarized to the effects of two adimensional parameters ${\varGamma }_{S}\equiv \frac{{d}_{S}}{{R}_{0}\sqrt{N}}$ and ${\varGamma }_{Y}\equiv \frac{{d}_{Y}}{{R}_{0}\sqrt{N}}$, which indicate the ratios of the maximum inhibition range and maximum induction range to the SAM size, respectively (${\varGamma }_{S}$ corresponds to $\varGamma$ in EDC2). Thus, the new model is determined by nine independent parameters: $N$, ${\varGamma }_{S}$, ${\alpha }_{S}$, ${A}_{S}$, ${B}_{S}$, ${\varGamma }_{Y}$, ${\alpha }_{Y}$, ${A}_{Y}$, and ${B}_{Y}$.

### SAM enlargement in model analysis

Douady and Couder’s previous work with DC2 (Douady and Couder 1996c) already dealt with the enlargement of SAM, by considering the time-dependent change of the ratio of the maximum inhibition range to the SAM size. This procedure is equivalent to describing ${\varGamma }_{S}$ as:

13 $${\varGamma }_{S}\left({t}_{sim}\right)\equiv \frac{{d}_{S}}{{R}_{0}\left({t}_{sim}\right)\sqrt{N}}=\frac{{{R}_{0}}_{f}}{{R}_{0}\left({t}_{sim}\right)}\frac{{d}_{S}}{{R}_{{0}_{f}}\sqrt{N}}={{\varGamma }_{S}}_{f}\frac{{{R}_{0}}_{f}}{{R}_{0}\left({t}_{sim}\right)},$$

where ${t}_{sim}$ is the time in the simulation normalized by ${V}_{0}/{R}_{0}$, ${{R}_{0}}_{f}$ indicates the final SAM size at ${t}_{sim}\to \infty$, and ${{\varGamma }_{S}}_{f}\equiv \frac{{d}_{S}}{{R}_{{0}_{f}}\sqrt{N}}$. The SAM enlargement was introduced as the function of ${\varGamma }_{S}\left({t}_{sim}\right)$:

14 $${\varGamma }_{S}\left({t}_{sim}\right)\equiv \frac{{{\varGamma }_{S}}_{i}+{{\varGamma }_{S}}_{f}}{2}-\frac{{{\varGamma }_{S}}_{i}-{{\varGamma }_{S}}_{f}}{2}\text{tanh}\left(\frac{{t}_{sim}-{t}_{i}}{\tau }\right),$$

where ${t}_{i}$ and $\tau$ represent the timing and slowness of the SAM enlargement, respectively. ${{\varGamma }_{S}}_{i}$ indicates the limit of ${\varGamma }_{S}\left({t}_{sim}\right)$ at ${t}_{sim}\to -\infty$.

We applied this way of dealing with the SAM enlargement to the new model, with introducing an additional parameter ${{\varGamma }_{Y}}_{f}$ to describe the effect from the time-dependent change of the SAM size on the inductive field. ${\varGamma }_{Y}\left({t}_{sim}\right)$ is defined with ${R}_{0}\left({t}_{sim}\right)$ and, by substituting it using Eq. 13, written as:

15 $${\varGamma }_{Y}\left({t}_{sim}\right)\equiv \frac{{d}_{Y}}{{R}_{0}\left({t}_{sim}\right)\sqrt{N}}=\frac{{{R}_{0}}_{f}}{{R}_{0}\left({t}_{sim}\right)}\frac{{d}_{Y}}{{R}_{{0}_{f}}\sqrt{N}}=\frac{{\varGamma }_{S}\left({t}_{sim}\right)}{{{\varGamma }_{S}}_{f}}{{\varGamma }_{Y}}_{f}.$$

### Computer simulations

Model simulations were implemented in C + + with Visual C + + in Microsoft Visual Studio® 2019 as an integrated development environment. Contour mapping was performed with OpenCV ver. 4.1.0 (https://opencv.org/).

Simulation analysis with EDC2 was performed using the codes previously published (Yonekura et al. 2019).

Computer simulations were initiated by placing a single primordium on the SAM periphery and performed with the spatial resolution of 0.1° All models were simulated with the time step of ${\Delta }{t}_{sim}=0.001$.

As a distance between points $\left({r}_{\left(1\right)}, \frac{\psi }{2}, {\theta }_{\left(1\right)}\right)$ and $\left({r}_{\left(2\right)}, \frac{\psi }{2}, {\theta }_{\left(2\right)}\right)$ on the conical surface, instead of the true Euclidian distance, its slightly modified version defined in the following equation is used to avoid the discontinuity problem (Douady and Couder 1996b).

16 $$d=\sqrt{\frac{{\left({r}_{\left(1\right)}-{r}_{\left(2\right)}\right)}^{2}}{N}+2 N{r}_{\left(1\right)}{r}_{\left(2\right)}\left(1-\text{cos}\left({\theta }_{\left(1\right)}-{\theta }_{\left(2\right)}\right)\right)}$$

In all model simulations, calculation was iterated while the total number of primordia was less than 100, $G$ was less than 5, and ${t}_{sim}$ was less than twice the number of primordia. Alternate and whorled patterns generated by simulation were judged for the stability and regularity of divergence angles and for the number of primordia per node, respectively. Then the patterns were categorized and displayed as shown in Fig. S1.

### Numerical solution

To solve equations numerically, C + + with Visual C + + in Microsoft Visual Studio® 2019 was used for calculations.

## Results

### Morphological characterization of phyllotaxis of *C. megalobractea*

To characterize morphologically real costoid phyllotaxis, we performed anatomical analysis of the shoot apex of *C. megalobractea* using materials collected from adult vegetative plants (Fig. 1b). Similarly to the other members of Costaceae (Hofmeister 1868; Snow 1952), *C. megalobractea* had obviously much smaller divergence angles than the divergence angles of ordinary phyllotactic spirals such as 137.5° (Fibonacci spiral) or 99.5° (Lucas spiral) (Jean 1994) (Fig. 1c). The mean value of the divergence angles in the mature plants of *C. megalobractea* was about 54°, which lies within the range of the divergence angles reported for *C. spicatus* (47.7°) by Snow (1952) and *C. scaber* (60°) and *C. cuspidatus* (73°) by Kirchoff and Rutishauser (1990). The natural log of the plastochron ratio $G$ was about 0.62 (Fig. 1c), which is very large compared to the $G$ values measured for distichous and spirodistichous patterns (Rutishauser 1998). In the seedlings of *C. megalobractea*, the direction of the phyllotactic spiral appears to be not fixed but random, for there were almost equal proportions of right-handed and left-handed spirals (Fig. S2).

### Construction of a new model of phyllotactic pattern formation

The small divergence angle of costoid phyllotaxis means that the radial position of each leaf primordium is close to that of its preceding primordium, and the large plastochron ratio suggests that the positioning of the incipient primordium on the SAM periphery is seemingly influenced by only its immediately preceding primordium among the existing primordia because older primordia are already too far away from the SAM periphery. Taking these features as they are, we hypothesized that there may be some inductive effect, in addition to the inhibitory effect, from the preceding leaf primordium on new primordium formation. Based on this hypothesis, we constructed a new mathematical model that assumes both inductive and inhibitory fields. In the new model, an inductive filed created by the inductive effect of each existing primordium is imposed on EDC2, and it is assumed that new primordium initiation is allowed only when the inductive and inhibitory field strengths are sufficiently high (larger than a given threshold) and sufficiently low (smaller than another given threshold), respectively.

### Generation of costoid phyllotaxis in computer simulation with the new model

**Fig. 2:**
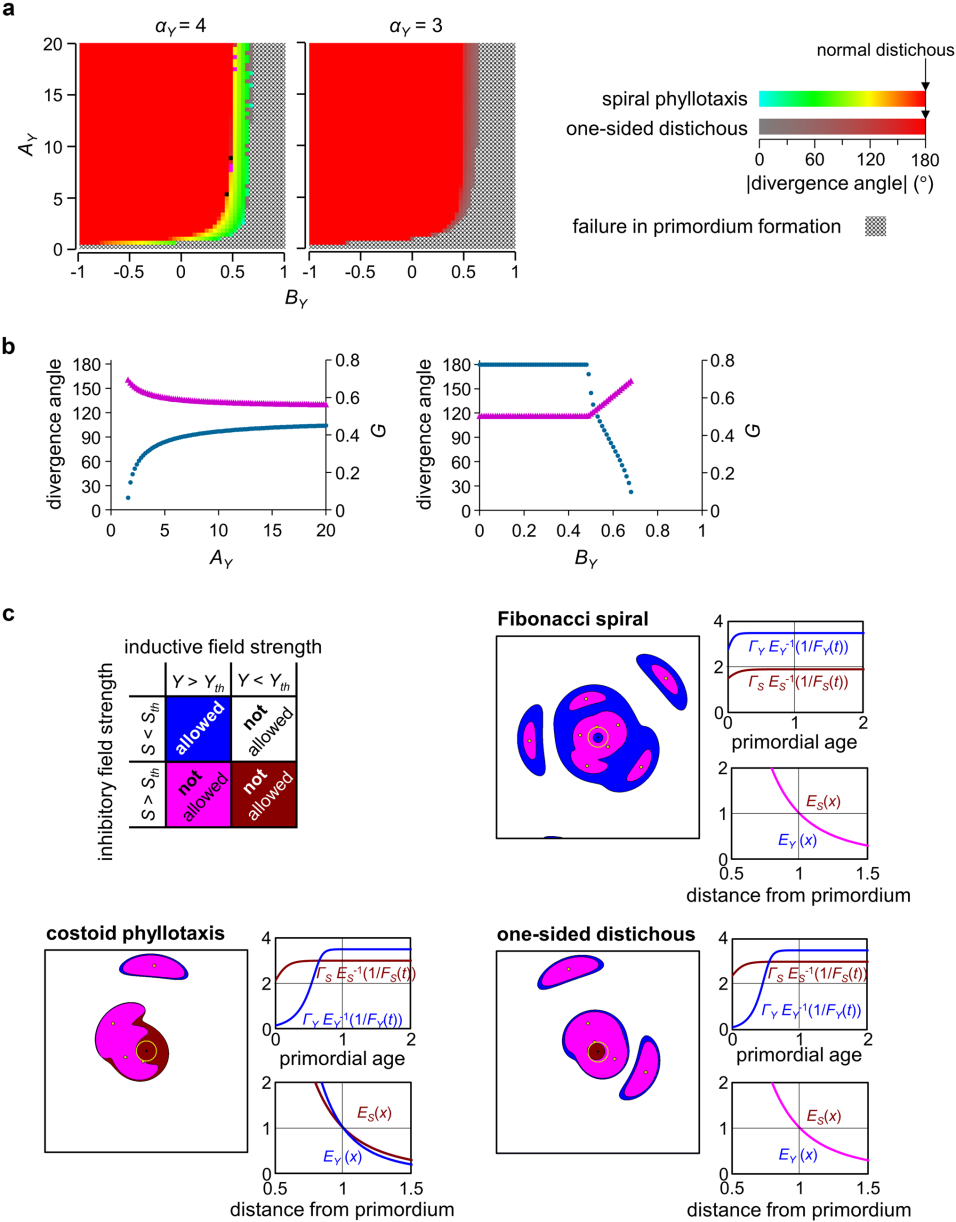
Generation of costoid and one-sided distichous patterns in computer simulations with the new model. **a** Distributions of phyllotactic patterns generated in the parameter space of the new model as influenced by ${\alpha }_{Y}$. Computer simulations using the new model were performed under various parameter settings for ${A}_{Y}$ and ${B}_{Y}$ (51 settings for $0\le {A}_{Y}\le 20$, 51 settings for $-1\le {B}_{Y}\le 1$) with a fixed set of the other parameters ($N=1/3$, ${\alpha }_{Y}=3$ or $4$, ${\alpha }_{S}=3$, ${\varGamma }_{Y}=3.5$, ${\varGamma }_{S}=3$, ${A}_{S}=10$, ${B}_{S}=0$). Simulations were started by placing a single primordium on the SAM periphery. Patterns obtained are displayed according to the color legend, of which simplified version is shown on the right side of this panel and its complete version is available in Supplementary Fig. S1. As all the phyllotactic patterns presented in this figure have a plastochron time ratio of about 1, the color of each pattern is simply determined by its divergence angle ratio, which is indicated here as the corresponding absolute value of the divergence angle. **b** Divergence angles and plastochron ratios of phyllotactic patterns generated in the new model as influenced by ${A}_{Y}$ and ${B}_{Y}$. Blue circles and magenta triangles represent values of the divergence angle and $G$, respectively. The absence of symbols means the failure in primordium formation. Computer simulations using the new model were performed under various parameter settings for ${A}_{Y}$ (101 settings for $0\le {A}_{Y}\le 20$ with ${B}_{Y}=0.55$) or for ${B}_{Y}$ (101 settings for $0\le {B}_{Y}\le 1$ with ${A}_{Y}=20$) with a fixed set of the other parameters ($N=1/3$, ${\alpha }_{Y}=4$, ${\alpha }_{S}=3$, ${\varGamma }_{Y}=3.5$, ${\varGamma }_{S}=3$, ${A}_{S}=10$, and ${B}_{S}=0$). **c** Three examples of phyllotactic patterns generated in computer simulations using the new model. The patterns are displayed as contour maps of the inductive field strength $Y$ and the inhibitory field strength $S$ within the shoot apical region. Yellow circle and yellow dot indicate the periphery of SAM and the center of each primordium, respectively. In the graphs, the age-dependent expansions of induction and inhibition ranges are shown by the curves of ${{\varGamma }_{Y}E}_{Y}^{-1}\left(1/{F}_{Y}\left(t\right)\right)$ and ${{\varGamma }_{S}E}_{S}^{-1}\left(1/{F}_{S}\left(t\right)\right)$, respectively, and the distance-dependent declines of inductive and inhibitory effects are shown by the curves of ${E}_{Y}\left(x\right)$ and ${E}_{S}\left(x\right)$, respectively. Upper right: Fibonacci spiral (parameters were set to $N=1/3$, ${\alpha }_{Y}=3$, ${\alpha }_{S}=3$, ${\varGamma }_{Y}=3.5$, ${\varGamma }_{S}=1.9$, ${A}_{Y}=20$, ${B}_{Y}=0$, ${A}_{S}=10$, and ${B}_{S}=0$). Lower left: costoid phyllotaxis ($N=1/3$, ${\alpha }_{Y}=4$, ${\alpha }_{S}=2$, ${\varGamma }_{Y}=3.5$, ${\varGamma }_{S}=3$, ${A}_{Y}=20$, ${B}_{Y}=0.64$, ${A}_{S}=10$, ${B}_{S}=0$). Lower right: one-sided distichous pattern ($N=1/3$, ${\alpha }_{Y}=3$, ${\alpha }_{S}=3$, ${\varGamma }_{Y}=3.5$, ${\varGamma }_{S}=3$, ${A}_{Y}=20$, ${B}_{Y}=0.52$, ${A}_{S}=10$, ${B}_{S}=0$). The upper left table indicates colors used for areas with different conditions for the inductive and inhibitory field strengths and whether primordium initiation is allowed or not allowed in these areas

Extensive computer simulations with the new model were conducted under a wide range of parameter combinations (Figs. S3–S8). As a result, we detected the occurrence of steep spirals in a particular range of conditions close to the conditions where no primordia were formed (Fig. 2a, b).

The relationships between the parameter conditions and the resultant patterns can be summarized as follows. Under such conditions that the increase of the inductive field emission is sufficiently fast and the inductive field strength is always larger than the threshold when and where the inhibitory field strength falls below the threshold, the patterns generated in the simulations with the new model are the same as generated by the EDC2 simulation (exemplified by the upper right panel of Fig. 2c, Video S1, and Fig. S9a–e). Under such conditions that the increase of the inductive field emission is very late and the inductive field strength is always smaller than the threshold when and where the inhibitory field strength falls below the threshold, no new primordium formation takes place. Under intermediate conditions in which the increase of the inductive field emission is moderately late, steep spirals are generated in some cases (lower left panel of Fig. 2c, Video S2, and Fig. S9f–j). These steep spirals were found to have not only small divergence angles but also large plastochron ratios (e.g., the divergence angle is 55.9° and $G$ is 0.682 for the spiral shown in the lower left panel of Fig. 2c and Video S2) and hence judged as representing costoid phyllotaxis.

We also found that it is necessary for the generation of the costoid pattern that the distance-dependent decline steepnesses of the inductive and inhibitory effects satisfy ${\alpha }_{Y}>{\alpha }_{S}$: otherwise, the delay of the increase of the inhibitory field emission resulted in the generation of the one-sided distichous pattern instead of the costoid patten (Fig. 2a, lower right panel of Fig. 2c, Video S3, and Fig. S10). One-sided distichy is a rare type of phyllotaxis, in which two orthostichies are not exactly opposite to each other but lie in one side.

**Fig. 3:**
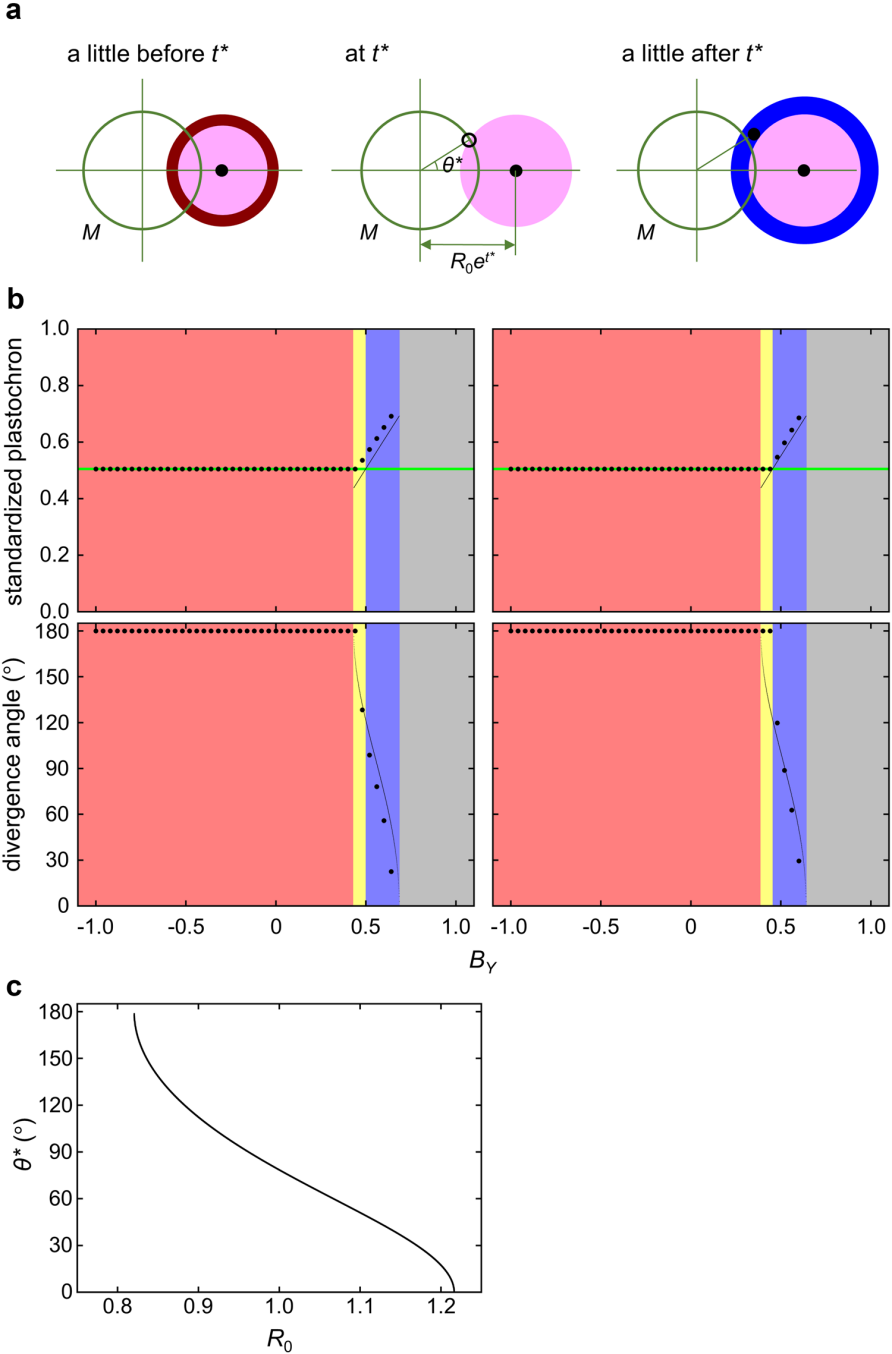
Theoretical analysis of requirements for the generation of costoid and one-sided distichous patterns. **a** Schematic illustration of the simplified situation of the new model. It is assumed that a new primordium arises when and where the effective inductive field from a single preceding primordium exceeds the effective inhibitory field from the same primordium on the circle $M$ representing the SAM periphery. Areas with different colors indicate different conditions for the relationship between the inductive and inhibitory filed strengths as shown in the upper left table of Fig. 2c. **b** Comparison between the solutions theoretically obtained from consideration of the simplified situation of the new model and the patterns generated by the computer simulations using the new model, under various ${B}_{Y}$ values ($-1\le {B}_{Y}\le 1$) with parameters of $N=1/3$, ${\alpha }_{Y}=3$ or $4$, ${\alpha }_{S}=3$, ${\varGamma }_{Y}=3.5$, ${\varGamma }_{S}=3$, ${A}_{Y}=20$, ${A}_{S}=10$, and ${B}_{S}=0$. Large dots show the standardized plastochrons ($G$) and divergence angles of the phyllotactic patterns generated by the computer simulations, while minute dots show the theoretical solutions for ${t}^{*}$ and ${\theta }^{*}$ if the solutions exist. Green horizontal lines in the upper panels indicate the level of ${G}_{S}$, which is the $G$ value calculated from the simulation without considering the inductive effect. Red zone: the range of ${B}_{Y}$ where the effective inductive field range always encompasses the effective inhibitory field range over $M$ and the solutions for ${t}^{*}$ and ${\theta }^{*}$ do not exist. Yellow zone: the range of ${B}_{Y}$ where the solutions for ${t}^{*}$ and ${\theta }^{*}$ exist and ${t}^{*}$ is smaller than ${G}_{S}$. Blue zone: the range of ${B}_{Y}$ where the solutions for ${t}^{*}$ and ${\theta }^{*}$ exist and ${t}^{*}$ is larger than ${G}_{S}$. Grey zone: the range of ${B}_{Y}$ where the effective inhibitory field range always encompasses the effective inductive field range over $M$ and the solutions for ${t}^{*}$ and ${\theta }^{*}$ do not exist. Left panels: ${\alpha }_{Y}$ was set to $4$. In the simulations with this ${\alpha }_{Y}$ setting, costoid phyllotaxis can occur under certain conditions in the yellow zone and any conditions in the blue zone. Right panels: ${\alpha }_{Y}$ was set to 3. In the simulations with this ${\alpha }_{Y}$ setting, one-sided distichous phyllotaxis can occur under any conditions in the blue zone. For details, see also Supplementary Text S1. **c** Relationship between the SAM size ${R}_{0}$ and the theoretically predicted divergence angle ${\theta }^{*}$ in the simplified situation with parameters of $N=1/3$, $\frac{{d}_{Y}}{\sqrt{N}}{\left(1+{e}^{-{A}_{Y}\left({t}^{*}-{B}_{Y}\right)}\right)}^{-\frac{1}{{\alpha }_{Y}}}=\frac{{d}_{S}}{\sqrt{N}}{\left(1+{e}^{-{A}_{S}\left({t}^{*}-{B}_{S}\right)}\right)}^{-\frac{1}{{\alpha }_{S}}}=3$, and $G=0.6$

From this finding, we postulated that it is necessary for costoid or one-sided distichous pattern formation that the inductive field strength rises above the threshold at some point on the SAM periphery where the inhibitory field strength is below the threshold (Fig. 3a). To identify the condition requirement theoretically, we considered a simplified situation of the new model in which only one primordium of the standardized age ${t}^{*}$ exists and both the inductive field strength and the inhibitory field strength reach their thresholds at the same point on the SAM periphery shifted by ${\theta }^{*}$ from the existing primordium in the angle direction. As a new primordium arises at this position at the very moment, ${\theta }^{*}$ and ${t}^{*}$ give the divergence angle and the standardized plastochron (correspondent to the natural log of the plastochron ratio, $G$), respectively. In the present situation, ${t}^{*}$ should satisfy the following equation (Supplementary Text S1):

17 $$\frac{{d_{Y} }}{{d_{S} }} = \frac{{\left( {1 + e^{{ - A_{Y} \left( {t^{*} - B_{Y} } \right)}} } \right)^{{\frac{1}{{\alpha _{Y} }}}} }}{{\left( {1 + e^{{ - A_{S} \left( {t^{*} - B_{S} } \right)}} } \right)^{{\frac{1}{{\alpha _{S} }}}} }},$$

where ${d}_{Y}$ and ${d}_{S}$ represent the maximum ranges of induction and inhibition by a single primordium, respectively. Thus, theoretically, parameter conditions for the existence of the solutions of Eq. 17 for ${t}^{*}$ are required for the generation of costoid and one-sided distichous patterns. Indeed, computer simulations showed that, only under these parameter conditions, the new model produced costoid or one-sided distichous phyllotaxis (Fig. 3b).

${\theta }^{*}$ can be calculated from ${t}^{*}$ according to the following relationship where ${R}_{0}$ represents the SAM size (Supplementary Text S1).

18 $$\theta * = {\text{cos}}^{{ - 1}} \left( {1 - \frac{1}{{2e^{{t*}} N}}\left( {\frac{{d_{Y} ^{2} }}{{R_{0} ^{2} }}\left( {1 + e^{{ - A_{Y} \left( {t* - B_{Y} } \right)}} } \right)^{{ - \frac{2}{{\alpha _{Y} }}}} - \frac{{\left( {e^{{t*}} - 1} \right)^{2} }}{N}} \right)} \right).$$

The theoretically determined values of ${t}^{*}$ and ${\theta }^{*}$ fit well to the $G$ values and the divergence angles of costoid and one-sided distichous phyllotaxes obtained in computer simulations (Fig. 3b). Of note, Eq. 18 also indicates that ${\theta }^{*}$ decreases as ${R}_{0}$ increases when all other parameters are fixed to be constant (Fig. 3c).

### Early changes in the divergence angle of costoid phyllotaxis in the new model

**Fig. 4:**
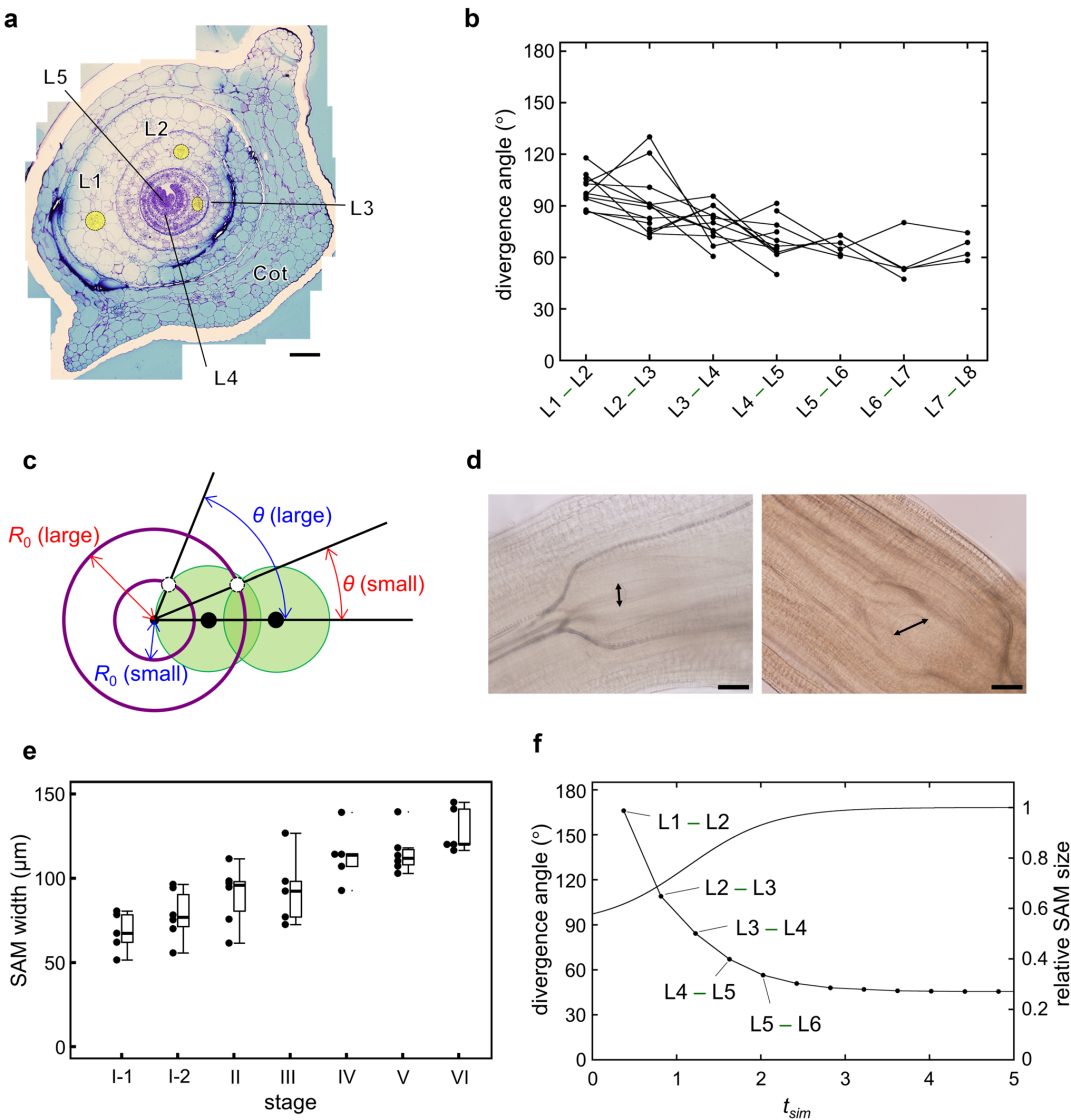
Early phyllotactic transition in the seedlings of *C. megalobractea*. **a** Transverse section of the shoot apex of a seedling. Leaves and leaf primordia are designated as ${L}_{1}$, ${L}_{2}$, ${L}_{3},$ etc., with ${L}_{1}$ being the oldest one. Cot indicates the cotyledon. For ${L}_{1}$ to ${L}_{3}$, the midveins are marked with yellow color. Bar: 200 μm. **b** Divergence angles measured from the transverse sections of the seedling shoot apices. Points linked by a line represent data from the same sample. **c** Schematic illustration of the negative correlation between the SAM size and the divergence angle. **d** Cleared shoot apex of the seedlings of *C. megalobractea*. Left: seedling shortly after germinated, which did not have visible true leaves. Right: seedling with five visible true leaves. Double arrows indicate the SAM width. Bars: 100 μm. **e**, Change in the SAM width during seedling development. Closed circles represent individual data. Boxes indicate quartiles, and whiskers indicate the minimum/maximum values among data whose difference from the lower/upper quartile is not more than the $1.5\times$ interquartile ranges. Developmental stages were determined by the number of visible leaves including a cotyledon. Stage I was divided into two sub-stages based on whether the cotyledon showed upward curling (stage I-1) or downward curling (stage I-2). **f**, Early phyllotactic transition of costoid phyllotaxis obtained from the computer simulation assuming SAM enlargement. The line chart shows the divergence angles between ${L}_{n}$ and ${L}_{n+1}$ against the timing of the emergence of ${L}_{n+1}$ expressed as the time in the simulation ${t}_{sim}$. The first true leaf primordium ${L}_{1}$ was set to emerge at ${t}_{sim}=0$. The curve indicates the ratio of the SAM size to the final SAM size ${{R}_{0}}_{f}$. Parameters were set to $N=1/3$, ${\alpha }_{Y}=4$, ${\alpha }_{S}=3$, ${\varGamma }_{Y}=1.79$, ${\varGamma }_{{S}_{i}}=3.2$, ${\varGamma }_{{S}_{f}}=1.71$, ${A}_{Y}=20$, ${B}_{Y}=0.3$, ${A}_{S}=10$, ${B}_{S}=0$, ${t}_{i}=0.82$, and $\tau =1$

To test the validity of the new model, we morphologically inspected seedlings of *C. megalobractea* for the early phyllotactic transition (Fig. 4a) and examined whether it agrees with the model prediction. As previously reported in the other species of Costaceae (Weisse 1932), the divergence angle of this plant decreased gradually from about 100º to about 60º on average during formation of five leaves (Fig. 4b).

From the negative correlation between the SAM size and the divergence angle in Eq. 18, the possibility arose that the decrease of the divergence angle in early leaves of the seedlings of *C. megalobractea* is associated with the increase of the SAM size (Fig. 4c), considering that the enlargement of SAM during seedling development has been often observed in many plants (Medford et al. 1992; Thompson et al. 2012). Measurement of the SAM width of seedlings of *C. megalobractea* at various stages indicated that indeed the SAM enlargement takes place during development (Fig. 4d, e).

Computer simulation with the new model assuming an increase in the SAM size comparable to the observed SAM enlargement could reproduce a decrease of the divergence angle in the costoid pattern, which was sharper than but not quite different from the real change of the divergence angle (Fig. 4f, Video S4). This result reinforced that the new model is plausible as a model of comprehensive generation of phyllotactic patterns including costoid phyllotaxis.

## Discussion

Based on the morphological features of costoid phyllotaxis, we hypothesized some effect of the existing leaf primordia to induce primordium initiation in their vicinity. In this study, we constructed a new mathematical model of phyllotaxis by introducing this hypothetical inductive effect into the generalized inhibitory-field model EDC2. Computer simulations using the new model succeeded in the generation of the costoid pattern, when the primordial age-dependent increase of the inductive field emission is moderately slow relative to the increase of the inhibitory field emission.

Notably, the new model can generate not only costoid phyllotaxis but also one-sided distichy in similar conditions. One-sided distichous phyllotaxis, characterized by its primordium initiation position on two angled orthostichies, is one of the rare types of phyllotaxis that have never been explained by the previously proposed mechanisms. The one-sided distichous pattern was reported for the inflorescence of *Thalia* and *Ctenanthe* (Marantaceae) and the rhizome of *Chamaecostus cuspidatus* (Costaceae) (Kirchoff 1986; Kirchoff and Rutishauser 1990; Schumann 1902; Schüepp 1928). All these plants belong to Zingiberales, as Costaceae plants exhibiting costoid phyllotaxis do. The fact that both costoid phyllotaxis and one-sided distichous phyllotaxis are exclusively found in Zingiberales implies some special relation between them. The adjacent occurrence of these uncommon phyllotaxes in the parameter space of the new model may account for their close relationship and restriction to Zingiberales and be regarded as supportive of the validity of the new model.

Theoretical considerations of the simplified situation of the new model in which only one preceding primordium exists revealed the principle of the mechanism working for the generation of the costoid and one-sided distichous patterns. According to this analysis, the costoid or one-sided distichous patterning requires that the induction range is encompassed by the inhibition range at first and later expands beyond the inhibition range somewhere on the SAM periphery. In this case, new primordium initiation takes place when and where the boundary of the induction range meets the boundary of the inhibition range on the SAM periphery.

Theoretical analysis also disclosed a negative relationship between the divergence angle and the SAM size in the costoid and one-sided distichous patterns generated in the new model. In agreement with this relationship, morphological analysis of seedlings of *C. megalobractea* showed that the divergence angle decreases as SAM becomes larger during development. Computer simulations further confirmed the realization of a similar phyllotactic transition under SAM enlargement in the new model. Collectively, these results suggest that the new model reflects the actual mechanism of leaf primordium positioning and that the inductive field hypothesized in it is truly involved in phyllotactic pattern formation and particularly important for costoid and one-sided distichous phyllotaxes in Zingiberales.

In spite of the drastic modification of the model with the introduction of the hypothetical inductive effect, there are limited changes in the distribution of phyllotactic patterns in the parameter space between EDC2 and the new model: substantially the only difference is the occurrence of the costoid and one-sided distichous patterns in the new model within a narrow range at the marginal conditions, outside which no primordium initiation is allowed. It may be the reason why plants having costoid phyllotaxis are restricted to Costaceae that, without luckily acquiring some strict regulation, plants in this narrow range of parameters would easily lose leaves by a small change in the parameters and be eliminated by natural selection.

Molecular biological studies have demonstrated that leaf primordium initiation is controlled by auxin and its polar transport (Reinhardt et al. 2000, 2003; Benková et al. 2003). This control was proposed to involve a positive feedback loop between auxin gradient and auxin polar transport by the auxin efflux carrier PIN1, which spontaneously creates auxin convergence, leading to leaf primordium initiation (Jönsson et al. 2006; Smith et al. 2006). In this scheme, the inhibitory effect of the existing primordia on the vicinal formation of a new primordium is correspondent to the auxin depletion by drainage into the existing auxin convergence from its vicinity (Mirabet et al. 2012). On the other hand, leaf primordia and young leaves are known to be major sources of auxin in the shoot apex (Cheng et al. 2007; Galvan-Ampudia et al. 2020). Furthermore, it was shown that the auxin biosynthesis enzyme YUCCA is essential for proper development of shoot lateral organs (Cheng et al. 2007) in Arabidopsis and that YUC1 and YUC4, major members of YUCCA, are expressed mainly in lateral organ primordia (Galvan-Ampudia et al. 2020). Most simply thinking from these roles of auxin, the possibility can be considered that, as well as the inhibitory effect, the inductive effect hypothesized in the new model may be mediated by auxin. Analysis of the dynamics of auxin biosynthesis and auxin transport with Costaceae would be important as the first step of the molecular characterization of costoid phyllotactic patterning and the new model.

### Supplementary Information

Below is the link to the electronic supplementary material.

Supplementary material 1 (PDF 5303.4 kb)

Fibonacci spiral generated in the new model. Movie of the upper right panel of Fig. 2c. Supplementary material 2 (AVI 5073.4 kb)

Costoid phyllotaxis generated in the new model. Movie of the lower left panel of Fig. 2c.Supplementary material 3 (AVI 3646.0 kb)

One-sided distichous generated in the new model. Movie of the lower right panel of Fig. 2c. Supplementary material 4 (AVI 4389.9 kb)

Early phyllotactic transition with SAM expansion. Movie of the computer simulation on which Fig. 4f was based. Yellow and gray circles show the current and initial SAM peripheries, respectively. Supplementary material 5 (AVI 3200.3 kb)

Source codes for the new model simulations as a compressed archive. Supplementary material 6 (7Z 7.5 kb)

Source codes for the new model simulations with the SAM enlargement as a compressed archive. Supplementary material 7 (7Z 3.3 kb)

## Data Availability

The data that support the findings of this study are available from the corresponding author upon reasonable request.
