# Supplementary material for: A new mathematical model of phyllotaxis to solve the genuine puzzle spiromonostichy
Source: J Plant Res. 2023 Oct 13;137(1):143–55. doi: 10.1007/s10265-023-01503-2 (PMC10764405; doi:10.1007/s10265-023-01503-2)
Supplement: Supplementary file 1 — Supplementary material 1 (PDF 5303.4 kb) [file 10265_2023_1503_MOESM1_ESM.pdf]

Journal of Plant Research:

A new mathematical model of phyllotaxis to solve the genuine puzzle spiromonostichy

Takaaki Yonekura*, Munetaka Sugiyama*
* Co-corresponding authors.

Address.
Department of Biological Science, Graduate School of Science, The University of Tokyo, Hongo 7-3-1, Bunkyo-ku, Tokyo 113-0033, Japan,

Email.
Takaaki Yonekura: t.yonekura@bs.s.u-tokyo.ac.jp
ORCID: 0000-0001-5428-8212
Munetaka Sugiyama: sugiyama@bs.s.u-tokyo.ac.jp
ORCID: 0000-0002-7050-8964

Supplementary Information (SI)

Text S1 and **Fig S1–S10** are included.

Text S1. Model-based theoretical analysis to identify requirements for the generation of the costoid and one-sided distichous patterns

Equations to be satisfied by the standardized plastochron and divergence angle

For theoretical analysis, we consider a simplified situation of the new model in which only one primordium of the standardized age t^* exists at $\left(R_0 e^{t^*}, \frac{\psi}{2}, 0\right)$ and both the inductive field strength and the inhibitory field strength reach their thresholds at the point $\left(R_0, \frac{\psi}{2}, \theta^*\right)$ on the circle M (Fig. 3a). That is, the boundary of the effective inductive field range (within which the inductive field strength is larger than the threshold Y_{th}) surrounding the single existing primordium meets the boundary of the effective inhibitory field range (within which the inhibitory field strength is larger than the threshold S_{th}) surrounding the same primordium on M. This situation places a new primordium arises at the position $\left(R_0, \frac{\psi}{2}, \theta^*\right)$ at the very moment, which means that t^* and θ^* correspond to the standardized plastochron and the divergence angle, respectively. The requirements for the existence of the solutions of t^* and θ^* should give the requirements for the generation of the costoid and one-sided distichous patterns.

The above situation can be described as follows by comparing Eqn 7 with Eqn 8 and using Eqn 9 to Eqn 12 in the main text:

$$\left(\frac{d(\theta^*)}{d_Y}\right)^{-\alpha_Y} \frac{1}{1+e^{-A_Y(t^*-B_Y)}} = \left(\frac{d(\theta^*)}{d_S}\right)^{-\alpha_S} \frac{1}{1+e^{-A_S(t^*-B_S)}} = 1. \qquad \text{(S1)}$$

On the other hand, $d(\theta^*)$ is calculated as the modified Euclidian distance between $\left(R_0, \frac{\psi}{2}, \theta^*\right)$ and $\left(R_0 e^{t^*}, \frac{\psi}{2}, 0\right)$ according to Eqn 16 in the main text:

$$d(\theta^*) = R_0 \sqrt{\frac{(1-e^{t^*})^2}{N} + 2Ne^{t^*}(1-\cos\theta^*)}. \qquad \text{(S2)}$$

Eqns S1, S2 indicate the relationships to be satisfied by t^* and θ^*.

Numerical determination of the standardized plastochron

By eliminating $d(\theta^*)$, Eqn 17 of the main text is derived from Eqn S1. The standardized plastochron t^* is obtained as the solution of Eqn 17. It is important to note that the t^* is determined independently of the meristem size R_0 and the shape N and is dependent on the

parameters characterizing the inhibitory and inductive effects.

Eqn 17 cannot be solved analytically for t^*. Before numerical solution, let us consider how many solutions Eqn 17 has from the extremum points of $f(t) \equiv \frac{\left(1+e^{-A_Y(t-B_Y)}\right)^{\frac{1}{\alpha_Y}}}{\left(1+e^{-A_S(t-B_S)}\right)^{\frac{1}{\alpha_S}}}$. At these extremum points,

$$\frac{d}{dt}f(t) = \frac{d}{dt}\left\{\frac{\left(1+e^{-A_Y(t-B_Y)}\right)^{\frac{1}{\alpha_Y}}}{\left(1+e^{-A_S(t-B_S)}\right)^{\frac{1}{\alpha_S}}}\right\}$$

$$= \frac{\left(1+e^{-A_Y(t-B_Y)}\right)^{\frac{1}{\alpha_Y}}}{\left(1+e^{-A_S(t-B_S)}\right)^{\frac{1}{\alpha_S}}} \cdot \frac{\alpha_Y A_S\left(1+e^{A_Y(t-B_Y)}\right) - \alpha_S A_Y\left(1+e^{A_S(t-B_S)}\right)}{\alpha_Y \alpha_S\left(1+e^{A_Y(t-B_Y)}\right)\left(1+e^{A_S(t-B_S)}\right)} = 0. \qquad \text{(S3)}$$

Because $\left(1+e^{-A_Y(t-B_Y)}\right)^{\frac{1}{\alpha_Y}}$, $\left(1+e^{-A_S(t-B_S)}\right)^{\frac{1}{\alpha_S}}$, α_Y, α_S, $\left(1+e^{A_Y(t-B_Y)}\right)$, and $\left(1+e^{A_S(t-B_S)}\right)$ are all positive, the solution(s) of Eqn S3 are equivalent to the solution(s) of the following equation:

$$\alpha_Y A_S\left(1+e^{A_Y(t-B_Y)}\right) - \alpha_S A_Y\left(1+e^{A_S(t-B_S)}\right) = 0. \qquad \text{(S4)}$$

At the extremum points of the left-side function of Eqn S5,

$$\frac{d}{dt}\left\{\alpha_Y A_S\left(1+e^{A_Y(t-B_Y)}\right) - \alpha_S A_Y\left(1+e^{A_S(t-B_S)}\right)\right\}$$

$$= A_Y A_S\left(\frac{\alpha_Y}{e^{A_Y B_Y}}e^{A_Y t} - \frac{\alpha_S}{e^{A_S B_S}}e^{A_S t}\right) = 0. \qquad \text{(S5)}$$

Eqn S6 can be solved for t as follows:

$$t = \frac{(A_S B_S - \ln\alpha_S) - (A_Y B_Y - \ln\alpha_Y)}{A_S - A_Y}. \qquad \text{(S6)}$$

Eqn S6 indicates that the left-side function of Eqn S4 has at most one extremum point and therefore that Eqn S3 has at most two solutions, which then indicates that $f(t)$ has at most two extremum points. These extremum point(s) can be determined numerically. Following this information, Eqn 17, which has the form of $f(t) = constant$, is shown to have at most three solutions, and finally t^* can be obtained by finding these solution(s) numerically.

Negative relationship between the SAM size and the divergence angle

From Eqn S1 and Eqn S2, we get the following equation:

$$d(\theta^*)^2 = \frac{{d_Y}^2}{\left(1+e^{-A_Y(t^*-B_Y)}\right)^{\frac{2}{\alpha_Y}}} = {R_0}^2\left(\frac{\left(1-e^{t^*}\right)^2}{N} + 2Ne^{t^*}(1-\cos\theta^*)\right). \qquad \text{(S7)}$$

Eqn 18 of the main text is derived from Eqns S7. As considered above, the standardized

plastochron t^* can be determined independently of the meristem size R_0 by solving Eqn 17 numerically. Therefore, Eqn 18 simply shows a negative relationship between the SAM size and the divergence angle, that is, θ^* becomes smaller when R_0 becomes larger under the condition that all other parameters are fixed to constant values (Fig. 3c).

Comparison between the results of theoretical analysis and of computer simulations

In Fig. 3b, the solutions of t^* and θ^* are plotted, if they exist, against B_Y, and are compared with G and the divergence angle of the patterns generated by the new model simulations with varying B_Y (Fig. 3b). This comparison demonstrates that the range of B_Y where the solutions of t^* and θ^* exist is almost correspondent to the B_Y range where the new model produced the costoid or one-sided distichous pattern and that, in this range, t^* and θ^* fit well to the G value and the divergence angle of the model-generated patterns, respectively. The good agreement validates our theoretical analysis with the simplified situation regarding the requirements for the generation of costoid or one-sided distichous pattern. Now the following explanation can be deduced. When B_Y is enough small, the induction range always encompasses the inhibition range, and in such case, common types of phyllotaxis are generated. When B_Y is moderately larger, the induction range is encompassed by the inhibition range at first and later it expands beyond the inhibition range somewhere on M, and in such case, costoid phyllotaxis or one-sided distichous patterns are generated. When B_Y is too large, the induction range is always encompassed by the inhibition range, and such case can never produce new primordia.

More accurately, however, there are some differences between the solutions obtained from theoretical analysis of the simplified situation and the simulation results. The t^* values are slightly smaller than the G values (upper panels of Fig. 3b), which should reflect the small effect from the second youngest primordium and/or older primordia. Moreover, the B_Y range generating costoid or one-sided distichous patterns in the simulation is a little different from the B_Y range for the existence of the solutions of t^* and θ^* (Fig. 3b). Such discrepancy is seen in the range of B_Y where t^* is smaller than G_S, the G value calculated from the simulation without considering the inductive effect (yellow zone in Fig. 3b), which is also attributable to the effect of the second youngest primordium and/or older primordium.

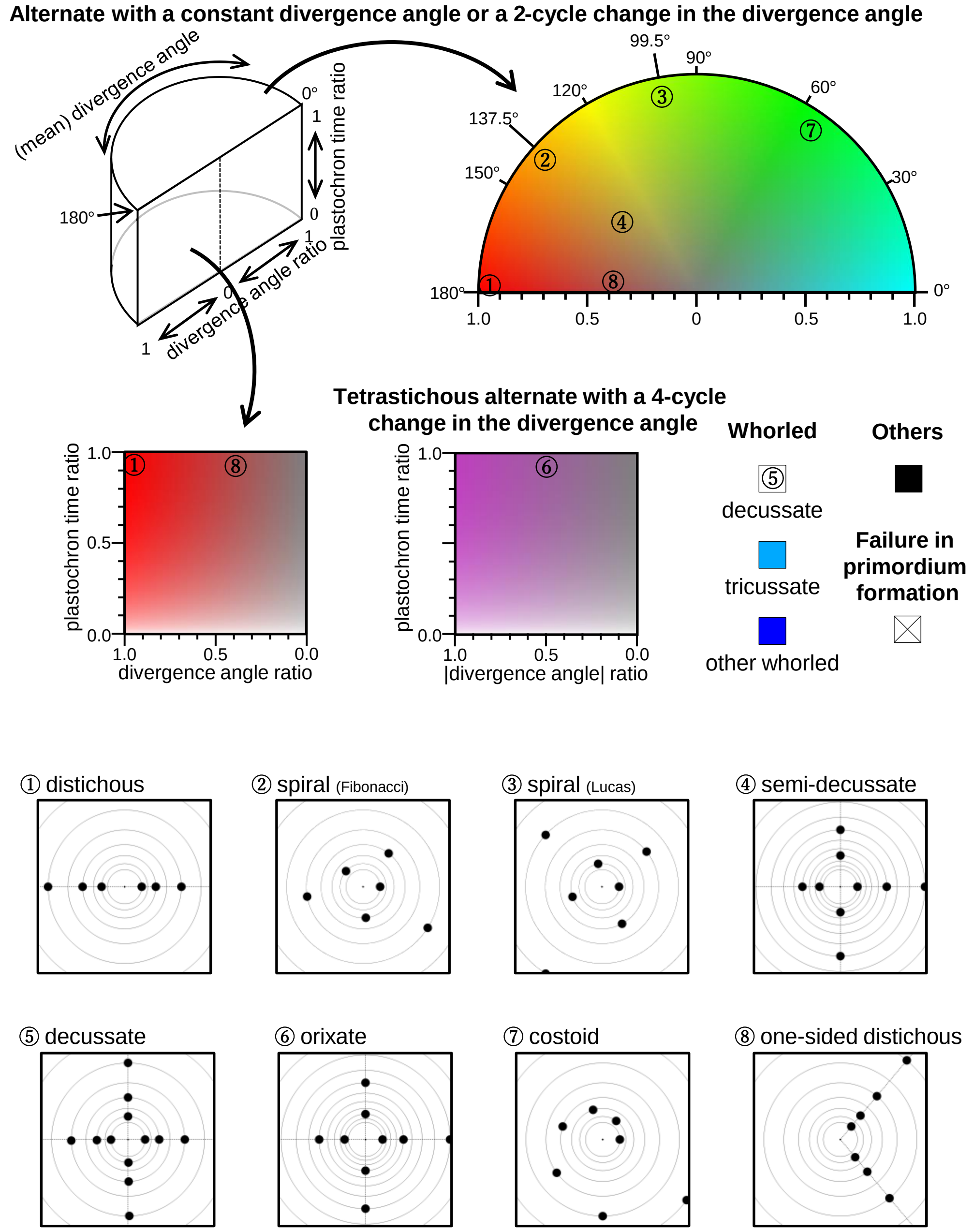

Fig. S1. Color legend for the phyllotactic patterns generated in computer simulations.
Phyllotactic patterns generated in computer simulations were classified into four categories: alternate patterns with a constant divergence angle or a two-cycle change in the divergence angle, tetrastichous alternate patterns with a four-cycle change in the divergence angle, whorled patterns, and other patterns. Whorled patterns were further classified into decussate, tricussate, and other whorled patterns. Different patterns are displayed by different colors in the parameter space of the model used for computer simulations. For regular alternate patterns with a constant divergence angle, the divergence angle is indicated by a color hue from cyan (0°) to red (180°). In the case of alternate patterns with a two-cycle divergence angle change with a constant absolute value of the divergence angle, the color hue is assigned for the absolute value of the divergence angle. In the case of other alternate patterns with a two-cycle divergence angle change, the color hue is assigned for the mean absolute value of the successive divergence angles. In these two-cycle alternate patterns, small-to-large ratios of two successive plastochrons and two successive divergence angles are represented by lightness (full lightness for 0) and saturation (full saturation for 1), respectively. Tetrastichous alternate patterns with a four-cycle divergence angle change are similarly expressed by color lightness and saturation based on their ratios of plastochron times and divergence angles; however, instead of the divergence angles themselves, the absolute values of divergence angles are used to calculate the ratio of divergence angles. As the divergence angle of this type of alternate pattern changes in the sequence of p, q, $-p$, and $-q$ ($-180° < p, q \leq 180°$), $|q|/|p|$ gives the ratio of the absolute values of divergence angles if $|p| > |q|$. When no new primordia were formed in simulation, it is indicated by the saltire mark (×). Typical examples of phyllotactic patterns are marked with circled numbers in the color legend and their schematic diagrams are shown at the bottom; 1. distichous, 2. Fibonacci spiral, 3. Lucas spiral, 4. semi-decussate, 5. decussate, 6. orixate, 7. costoid, and 8. one-sided distichous. The color assignment is modified from Yonekura et al., (2019).

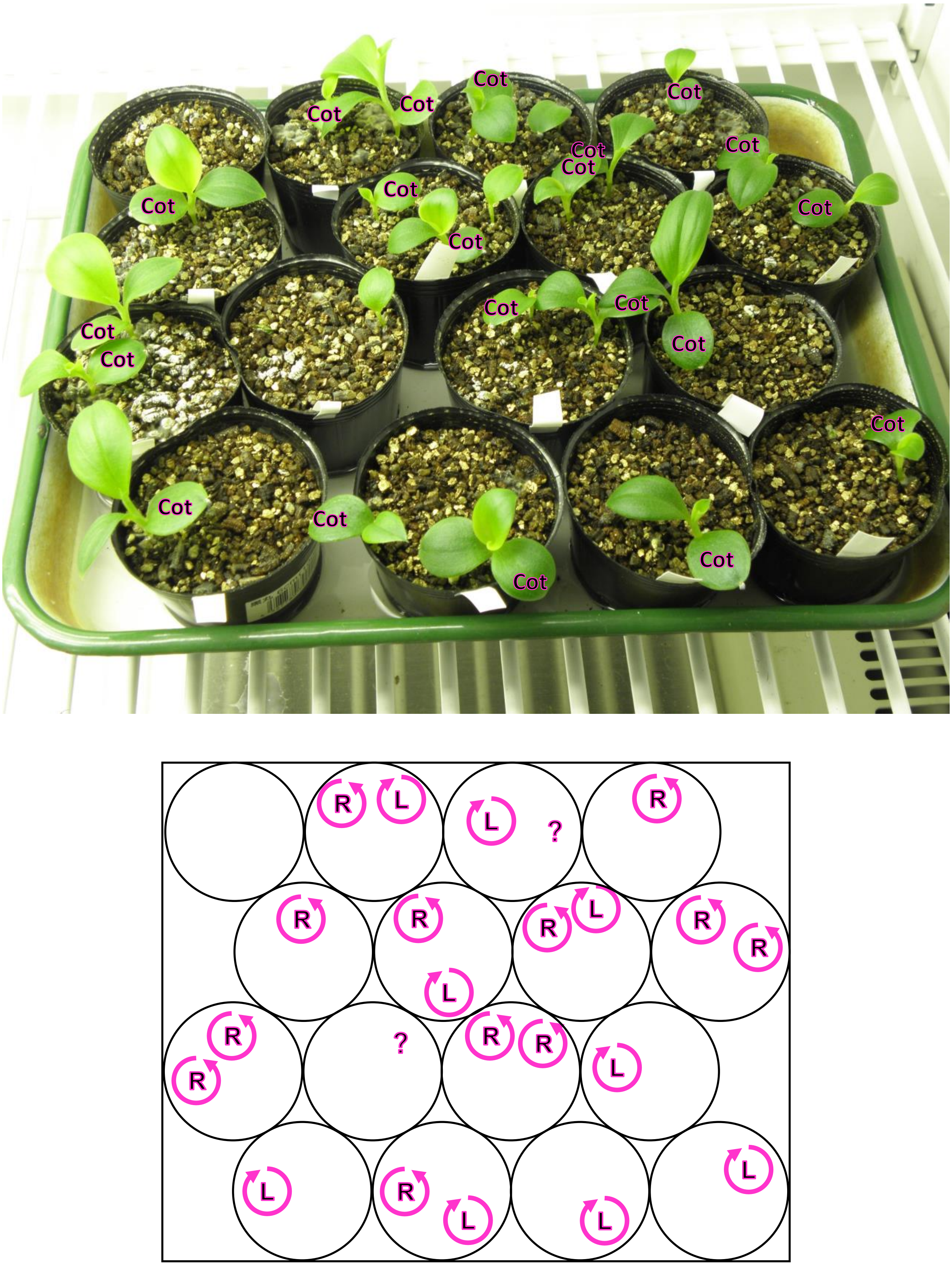

Fig. S2. Phyllotactic spiral directions in *C. megalobractea* seedlings

The upper panel shows a photograph of 37 days-after-sowing seedlings of *C. megalobractea* and the lower panel indicates their phyllotactic spiral directions. Cot, L, and R represent cotyledon, left-handed, and right-handed, respectively. Among these seedlings, 9 had a left-handed spiral while 12 had a right-handed spiral. The bias in the spiral direction from the 1:1 ratio is not significant (p = 0.67, binomial test).

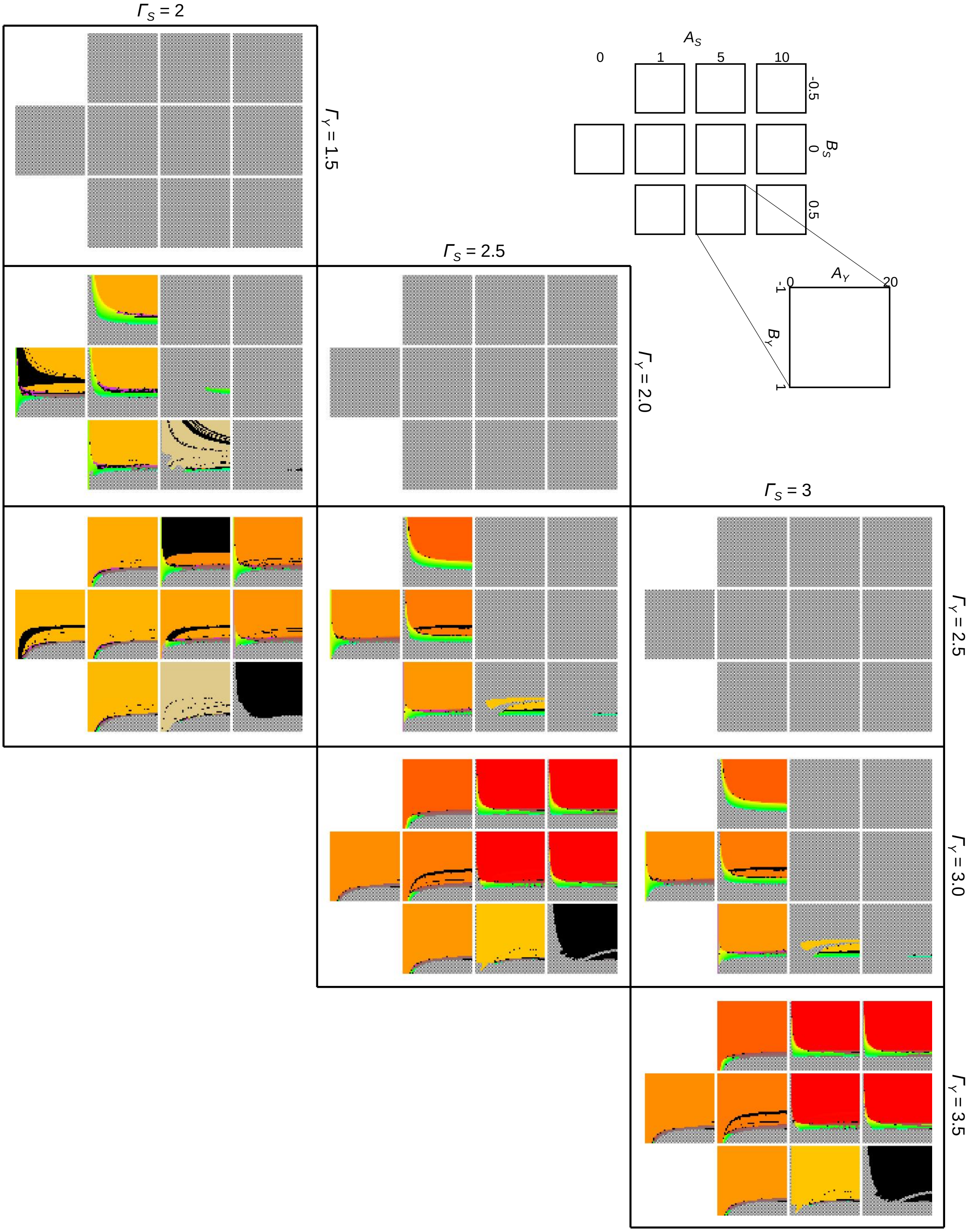

Fig. S3. Computer simulations with the new model over a wide range of combinations of seven parameters focusing on the inductive effect (1)

Computer simulations were performed using the new model under various settings of six parameters: 51 settings for A_Y ($0 \leq A_Y \leq 20$), 51 settings for B_Y ($-1 \leq B_Y \leq 1$), 9 settings for Γ_Y and Γ_S ($(\Gamma_Y, \Gamma_S) = (1.5, 2)$, $(2, 2)$, $(2.5, 2)$, $(2, 2.5)$, $(2.5, 2.5)$, $(3, 2.5)$, $(2.5, 3)$, $(3, 3)$, or $(3, 3.5)$), 4 settings for A_S ($A_S = 0$, 1, 5, or 10), and 3 settings for B_S ($B_S = 0$, -0.5, or 0.5). N, α_Y, and α_S were fixed to 1/3, 4, and 3, respectively. The patterns obtained are displayed in the A_Y B_Y space according to the color legend shown in Fig 2. Simulations were started by placing a single primordium.

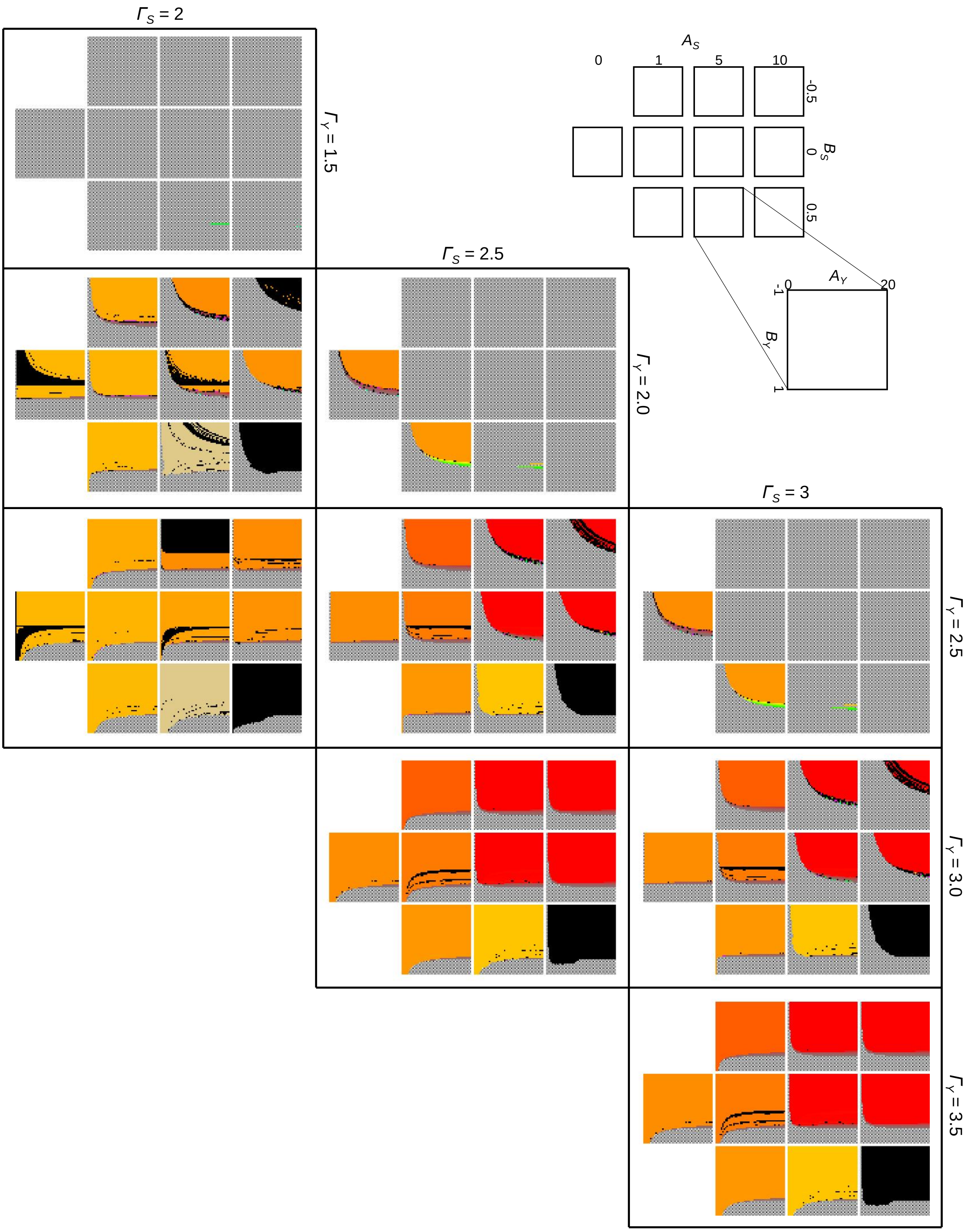

Fig. S4. Computer simulations with the new model over a wide range of combinations of seven parameters focusing on the inductive effect (2)

Computer simulations were performed using the new model under various settings of six parameters: 51 settings for A_Y ($0 \leq A_Y \leq 20$), 51 settings for B_Y ($-1 \leq B_Y \leq 1$), 9 settings for Γ_Y and Γ_S ($(\Gamma_Y, \Gamma_S) = (1.5, 2)$, $(2, 2)$, $(2.5, 2)$, $(2, 2.5)$, $(2.5, 2.5)$, $(3, 2.5)$, $(2.5, 3)$, $(3, 3)$, or $(3, 3.5)$), 4 settings for A_S ($A_S = 0$, 1, 5, or 10), and 3 settings for B_S ($B_S = 0$, -0.5, or 0.5). N, α_Y, and α_S were fixed to 1/3, 3, and 3, respectively. The patterns obtained are displayed in the A_Y B_Y space according to the color legend shown in Fig 2. Simulations were started by placing a single primordium.

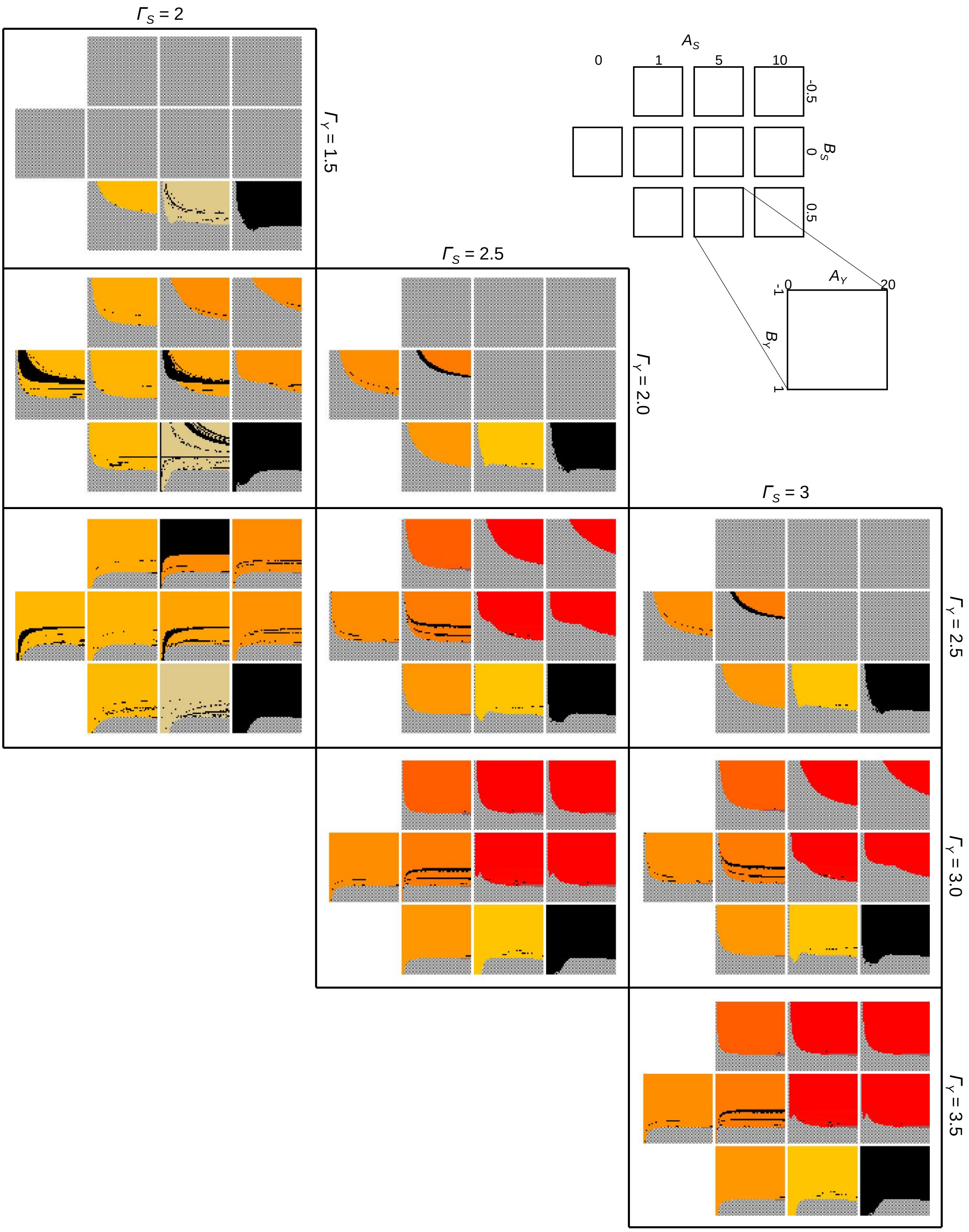

Fig. S5. Computer simulations with the new model over a wide range of combinations of seven parameters focusing on the inductive effect (3)

Computer simulations were performed using the new model under various settings of six parameters: 51 settings for A_Y ($0 \leq A_Y \leq 20$), 51 settings for B_Y ($-1 \leq B_Y \leq 1$), 9 settings for Γ_Y and Γ_S ($(\Gamma_Y, \Gamma_S) = (1.5, 2)$, $(2, 2)$, $(2.5, 2)$, $(2, 2.5)$, $(2.5, 2.5)$, $(3, 2.5)$, $(2.5, 3)$, $(3, 3)$, or $(3, 3.5)$), 4 settings for A_S ($A_S = 0$, 1, 5, or 10), and 3 settings for B_S ($B_S = 0$, -0.5, or 0.5). N, α_Y, and α_S were fixed to 1/3, 2, and 3, respectively. The patterns obtained are displayed in the A_Y B_Y space according to the color legend shown in Fig 2. Simulations were started by placing a single primordium.

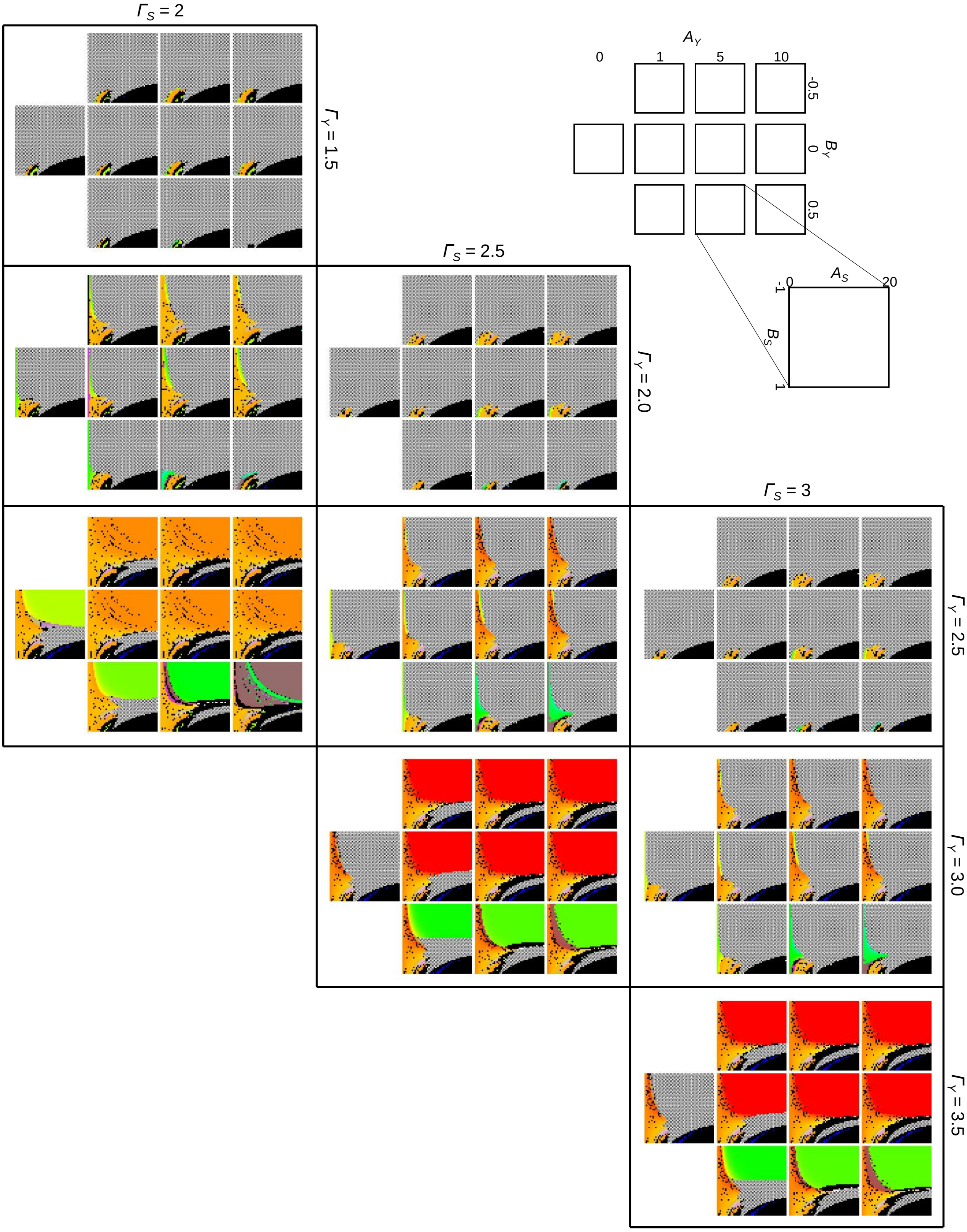

Fig. S6. Computer simulations with the new model over a wide range of combinations of seven parameters focusing on the inhibitory effect (1)

Computer simulations were performed using the new model under various settings of six parameters: 51 settings for A_S ($0 \leq A_S \leq 20$), 51 settings for B_S ($-1 \leq B_S \leq 1$), 9 settings for Γ_Y and Γ_S ($(\Gamma_Y, \Gamma_S) = (1.5, 2)$, $(2, 2)$, $(2.5, 2)$, $(2, 2.5)$, $(2.5, 2.5)$, $(3, 2.5)$, $(2.5, 3)$, $(3, 3)$, or $(3, 3.5)$), 4 settings for A_Y ($A_Y = 0$, 1, 5, or 10), and 3 settings for B_Y ($B_Y = 0$, -0.5, or 0.5). N, α_Y, and α_S were fixed to $1/3$, 4, and 3, respectively. The patterns obtained are displayed in the $A_S\,B_S$ space according to the color legend shown in Fig 2. Simulations were started by placing a single primordium.

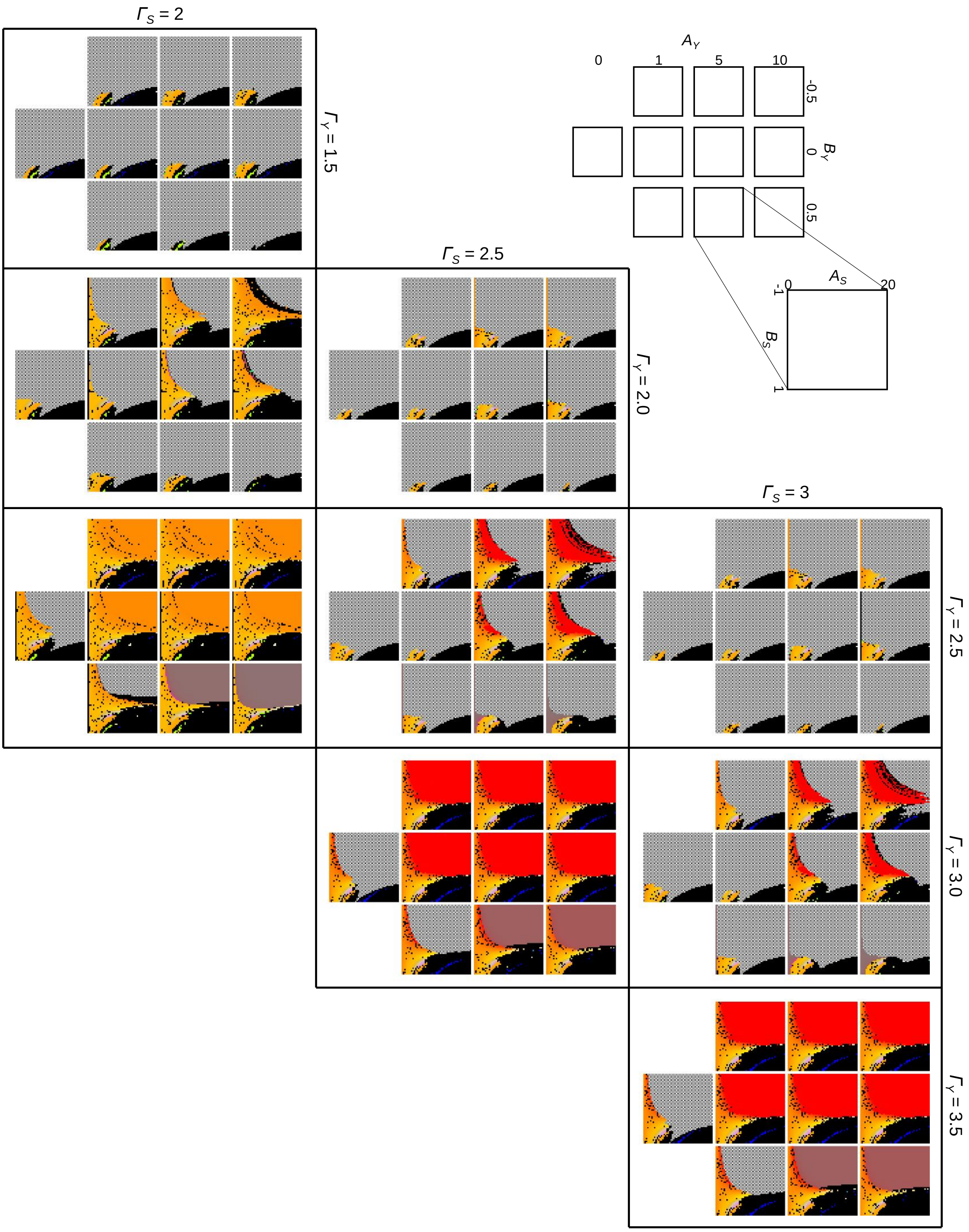

Fig. S7. Computer simulations with the new model over a wide range of combinations of seven parameters focusing on the inhibitory effect (2)

Computer simulations were performed using the new model under various settings of six parameters: 51 settings for A_S ($0 \leq A_S \leq 20$), 51 settings for B_S ($-1 \leq B_S \leq 1$), 9 settings for Γ_Y and Γ_S ($(\Gamma_Y, \Gamma_S) = (1.5, 2)$, $(2, 2)$, $(2.5, 2)$, $(2, 2.5)$, $(2.5, 2.5)$, $(3, 2.5)$, $(2.5, 3)$, $(3, 3)$, or $(3, 3.5)$), 4 settings for A_Y ($A_Y = 0$, 1, 5, or 10), and 3 settings for B_Y ($B_Y = 0$, -0.5, or 0.5). N, α_Y, and α_S were fixed to 1/3, 3, and 3, respectively. The patterns obtained are displayed in the A_S B_S space according to the color legend shown in Fig 2. Simulations were started by placing a single primordium.

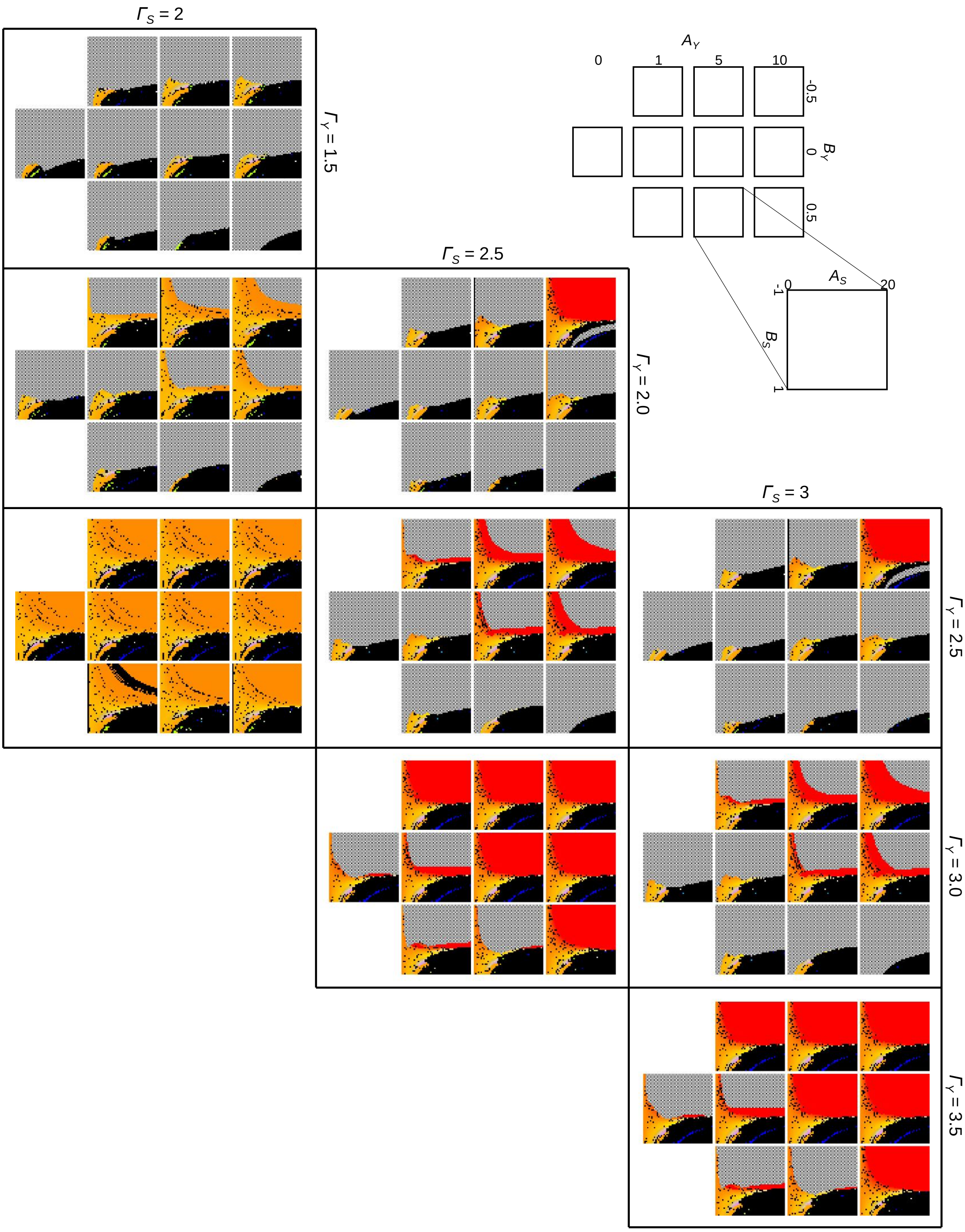

Fig. S8. Computer simulations with the new model over a wide range of combinations of seven parameters focusing on the inhibitory effect (3)

Computer simulations were performed using the new model under various settings of six parameters: 51 settings for A_S $(0 \leq A_S \leq 20)$, 51 settings for B_S $(-1 \leq B_S \leq 1)$, 9 settings for Γ_Y and Γ_S $((\Gamma_Y, \Gamma_S) = (1.5, 2), (2, 2), (2.5, 2), (2, 2.5), (2.5, 2.5), (3, 2.5), (2.5, 3), (3, 3)$, or $(3, 3.5))$, 4 settings for A_Y $(A_Y = 0, 1, 5$, or $10)$, and 3 settings for B_Y $(B_Y = 0, -0.5$, or $0.5)$. N, α_Y, and α_S were fixed to $1/3$, 2, and 3, respectively. The patterns obtained are displayed in the A_S B_S space according to the color legend shown in Fig 2. Simulations were started by placing a single primordium.

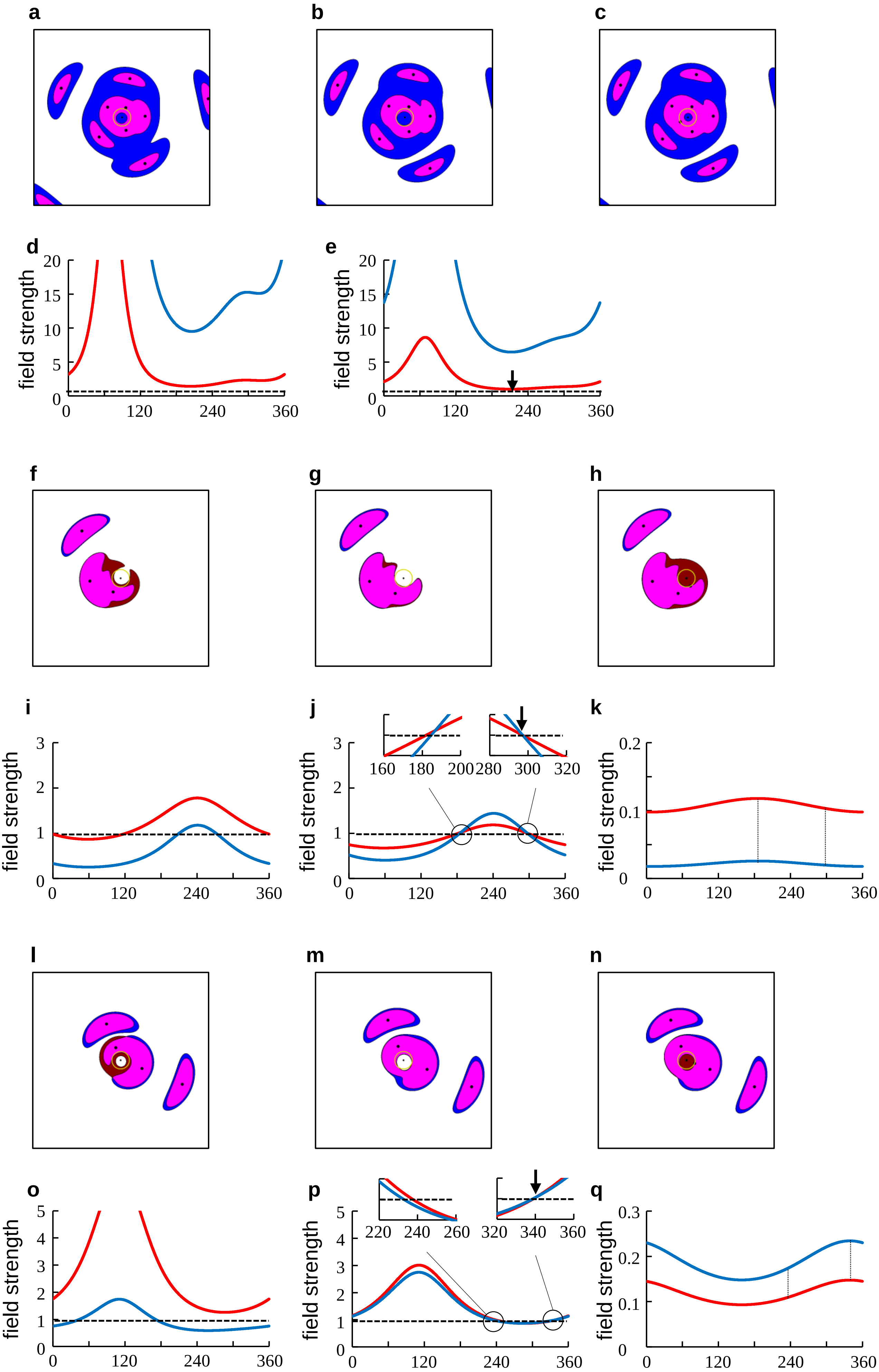

a
b
c
d
e
f
g
h
i
j
k
l
m
n
o
p
q
field strength
0
5
10
15
20
0
120
240
360
1
2
3
0.1
0.2
0.3
4
160
180
200
220
260
280
300
320
340

Fig. S9. Inductive and inhibitory field profiles at the SAM periphery as influenced by α_Y and α_S

a–**e**, Fibonacci spiral generated by the new model under $N = 1/3$, $\alpha_Y = 3$, $\alpha_S = 3$, $\Gamma_Y = 3.5$, $\Gamma_S = 1.9$, $A_Y = 20$, $B_Y = 0$, $A_S = 10$, and $B_S = 0$. **f**–**k**, Costoid phyllotaxis generated by the new model under $N = 1/3$, $\alpha_Y = 4$, $\alpha_S = 2$, $\Gamma_Y = 3.5$, $\Gamma_S = 3$, $A_Y = 20$, $B_Y = 0.64$, $A_S = 10$, and $B_S = 0$. **l**–**q**, One-sided distichous phyllotaxis generated by the new model under $N = 1/3$, $\alpha_Y = 3$, $\alpha_S = 3$, $\Gamma_Y = 3.5$, $\Gamma_S = 3$, $A_Y = 20$, $B_Y = 0.52$, $A_S = 10$, and $B_S = 0$. The contour maps 0.1 standardized time unit before (**a**, **f**, **l**), immediately before (**b**, **g**, **m**), and immediately after a new primordium arises (**c**, **h**, **n**) are shown. The blue, brown, and pink areas represent the region where the inductive field strength exceeds a given threshold, the region where the inhibitory field strength exceeds a given threshold, and the region where both the inductive and inhibitory field strengths exceed given thresholds, respectively. The inductive (blue) and inhibitory (red) field strengths on the circle M 0.1 standardized time unit before (**d**, **i**, **o**) and immediately before (**e**, **j**, **p**) a new primordium arises are shown with angles as abscissa. Dashed line indicates the threshold level (field $\text{strength} = 1$), and arrows point the positions which a new primordium can arise, that is, the inductive field strength exceeds a given threshold while the inhibitory field strength falls below a given threshold. In **j** and **p**, there are two positions where the inductive and inhibitory field strengths are almost equivalent and also close to the thresholds. Which of these positions is chosen for the site of new primordium formation is determined by the effect of the second youngest primordium P2. **k** and **q** show only the inductive (blue) and inhibitory (red) field strengths derived from P2. The vertical dotted lines indicate the substructions between the inductive and inhibitory effects of P2 at the positions where the inhibitory field strength is equivalent to its given threshold. In **k,** as the angle-dependent change of the P2 effect is larger in inhibition than in induction on the circle M due to $\alpha_Y > \alpha_S$, the position far from P2 receives a smaller inhibitory effect from P2 than the position close to P2 and is therefore chosen as the site of new primordium formation, resulting the generation of costoid phyllotaxis. In **q**, as the angle-dependent change of the P2 effect is larger in induction than in inhibition on the circle M due to $\alpha_Y \leq \alpha_S$, the position close to P2 receives a larger inductive effect from P2 than the position far from P2 and is therefore chosen as the site of new primordium formation, resulting the generation of one-sided phyllotaxis phyllotaxis.

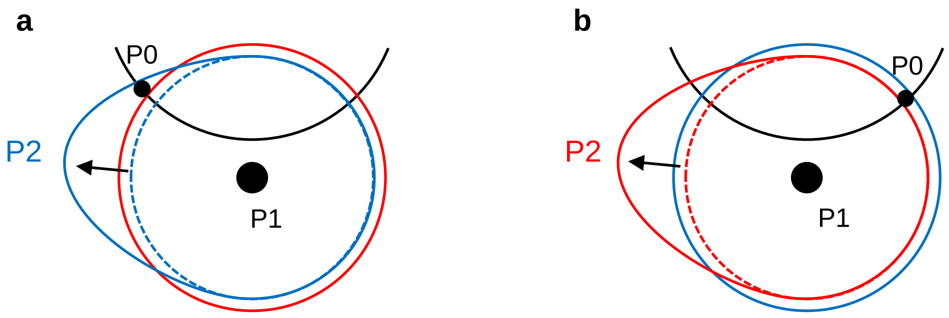

Fig. S10. Difference of the induction and inhibition ranges between conditions that produce one-sided distichous and costoid phyllotaxes

Schematic views of the difference of the induction and inhibition ranges between parameter conditions that produce one-sided distichous and costoid phyllotaxes at the time of the initiation of a new primordium (P0). Blue and red lines show the induction and inhibition ranges, respectively. Dashed lines indicate the induction/inhibition ranges of P1 without considering the effects of the older primordium, whereas solid lines indicate the induction/inhibition range considering the effect of P2. When $\alpha_Y \leq \alpha_S$ (**a**), the effect of P2 is more “inductive” and P0 arises at the side close to P2, leading to one-sided distichy. When $\alpha_Y > \alpha_S$ (**b**), the effect of P2 is more “inhibitory” and P0 arises at the side far from P2, leading to costoid phyllotaxis.
